# Automated prediction of the clinical impact of structural copy number variations

**DOI:** 10.1038/s41598-021-04505-z

**Published:** 2022-01-11

**Authors:** M. Gažiová, T. Sládeček, O. Pös, M. Števko, W. Krampl, Z. Pös, R. Hekel, M. Hlavačka, M. Kucharík, J. Radvánszky, J. Budiš, T. Szemes

**Affiliations:** 1 Geneton Ltd, 84104 Bratislava, Slovakia; 2 Department of Computer Science, Faculty of Mathematics, Physics and Informatics, Comenius University, 84248 Bratislava, Slovakia; 3 Department of Molecular Biology, Faculty of Natural Sciences, Comenius University, 84215 Bratislava, Slovakia; 4 Institute of Clinical and Translational Research, Biomedical Research Center, Slovak Academy of Sciences, 84505 Bratislava, Slovakia; 5 Comenius University Science Park, 84104 Bratislava, Slovakia; 6 Slovak Center of Scientific and Technical Information, 81104 Bratislava, Slovakia

**Keywords:** Machine learning, Structural variation

## Abstract

Copy number variants (CNVs) play an important role in many biological processes, including the development of genetic diseases, making them attractive targets for genetic analyses. The interpretation of the effect of these structural variants is a challenging problem due to highly variable numbers of gene, regulatory, or other genomic elements affected by the CNV. This led to the demand for the interpretation tools that would relieve researchers, laboratory diagnosticians, genetic counselors, and clinical geneticists from the laborious process of annotation and classification of CNVs. We designed and validated a prediction method (ISV; Interpretation of Structural Variants) that is based on boosted trees which takes into account annotations of CNVs from several publicly available databases. The presented approach achieved more than 98% prediction accuracy on both copy number loss and copy number gain variants while also allowing CNVs being assigned “uncertain” significance in predictions. We believe that ISV’s prediction capability and explainability have a great potential to guide users to more precise interpretations and classifications of CNVs.

## Introduction

Copy number variants (CNVs) are unbalanced structural rearrangements of the genome leading to genetic and phenotypic variability between individuals and populations. It includes gains or losses of particular DNA sequences that may contribute to the development of human genetic diseases^1,2^ including microdeletion syndromes such as DiGeorge (22q11.2), Wolf-Hirschhorn (4p16.3), Prader-Willi and Angelman 15q11, Cri-Du-Chat (5p15), or 1p36 deletion^3^. It is known that CNVs can directly affect the gene coding sequence and cause disruption of a gene or alter gene dosage^4^. It was also shown that CNVs can affect gene expression indirectly. They have the potential to disrupt the spatial organization of the genome, by altering chromatin interaction domains^5–7^. Other molecular mechanisms by which CNVs may influence gene expression are through harboring the sequence of non-coding RNAs^8^, unmasking of recessive mutations, or functional polymorphisms when a copy number loss occurs^9^.

Various methods have been developed for the analysis of CNVs, from conventional cytogenetic methods, through microarrays to next-generation sequencing (NGS)^1,10^. In recent years, NGS has become a valuable tool for clinical diagnostics and represents a sensitive and accurate approach for the detection of CNVs with a wide range of sizes. The decreasing cost and widening deployment of NGS in the clinical area lead to a continuous increase in the number of identified variants^11^. This method has enabled genome-wide detection of CNVs in clinically affected individuals, as well as in the general population^11,12^. Due to significant progress in the detection of structural variants, we are now able to detect thousands of structural variants with a deep coverage sequencing in a human genome. However, since the speed of novel variant identification is far greater than the speed of their interpretation, there is a growing gap in our understanding of the clinical implications of DNA variants^11^.

In the past, the prediction of the impact of single nucleotide polymorphisms on the protein function met a similar problem, and great effort led to the development of many tools for pathogenicity prediction^13^. Today, some of these tools can calculate a score of pathogenicity for variants located in various positions throughout the genome. However, the development of such tools for structural variants seems to be more difficult. This is because CNVs have a wide spectrum of lengths, ranging from 50 bp to several Mbp. The length is an issue mainly because of uneven distribution of genomic content, meaning that a small CNV overlapping an important gene will likely be more harmful than a large CNV in an element void region. Moreover, the genomic coordinates highly differ, affecting various genes, regulatory, or other functionally important regions. These factors should be considered when developing a method for predicting the impact of structural variants for appropriate prioritization and classification of such variants^14^.

In 2019, an ACMG scheme was developed for the interpretation of CNVs^15^ to standardize and help with evaluations of the pathogenicity of CNVs. The scheme takes into account gene annotations and known regulatory, benign, or conserved regions which are overlapped by a given CNV. The CNV is then classified with standard five-tier classification (pathogenic, likely pathogenic, uncertain significance, likely benign, benign). Multiple tools have adopted these standards and are publicly available, such as ClassifyCNV^16^ or AnnotSV^17^. The tools differ in the usage of data sources, evaluation, and subsequent rating (classification) of clinical significance. The ACMG scheme classifies CNVs with great accuracy, however, at the cost of assigning most CNVs to the uncertain significance class. The ClassifyCNV performance could be used as an example of such a conservative classification. When evaluating benign/likely benign ClinVar CNVs the tool provides 99.6% specificity (concordance between the ClinVar classification and the ClassifyCNV result), but the sensitivity was low (11.8%), thus the majority of benign variants were classified as variants of uncertain significance^16^. In addition, automation of the entire evaluation of the ACMG scheme is impossible without further input from physicians, especially in evaluating patterns of family history inheritance.

SVScore^14^ was one of the first methods to directly produce pathogenicity scores for CNVs by aggregating per-base single nucleotide polymorphism pathogenicity scores from CADD v1.3 (Combined Annotation Dependent Depletion)^18^. Several machine learning-based tools have been proposed for the interpretation of CNVs as well. StrVCTVRE^19^ focuses on exonic CNVs. The authors trained a random forest classifier utilizing features describing gene importance, coding regions, conservation, expression, and exon structure. The model achieves a Receiver Operator Characteristic–Area Under Curve (ROC–AUC) score of 0.823, which is an improvement over SVScore’s performance (ROC–AUC = 0.71)^19^. SVFX^20^ focuses mainly on cancer-causing structural variants and treats somatic and germline CNVs separately by training a classifier for both cases. The pathogenicity score is derived “by comparing the genomic and tissue-specific epigenomic features of a given SV with those of known benign structural variants”. According to the publication, the somatic model should achieve ROC–AUC scores of 0.865 and 0.835 for deletions and duplications respectively. The germline model achieved a ROC–AUC score of 0.8^20^.

In the present study, we demonstrate that the clinical impact of copy number variation can be predicted reliably and with high accuracy using machine learning to help researchers, laboratory diagnosticians, genetic counselors, and clinical geneticists with the interpretation process. Given a set of CNV coordinates specified by the first and the last affected base on the chromosome, and its type (either copy number loss or copy number gain), we propose a machine learning-based approach for the task of CNV’s pathogenicity prediction—ISV (Interpretation of Structural Variants). We describe the CNV annotation process, generation of training, validation, and test sets, the machine learning procedure, and description of evaluation data.

## Materials and methods

**Figure 1:**
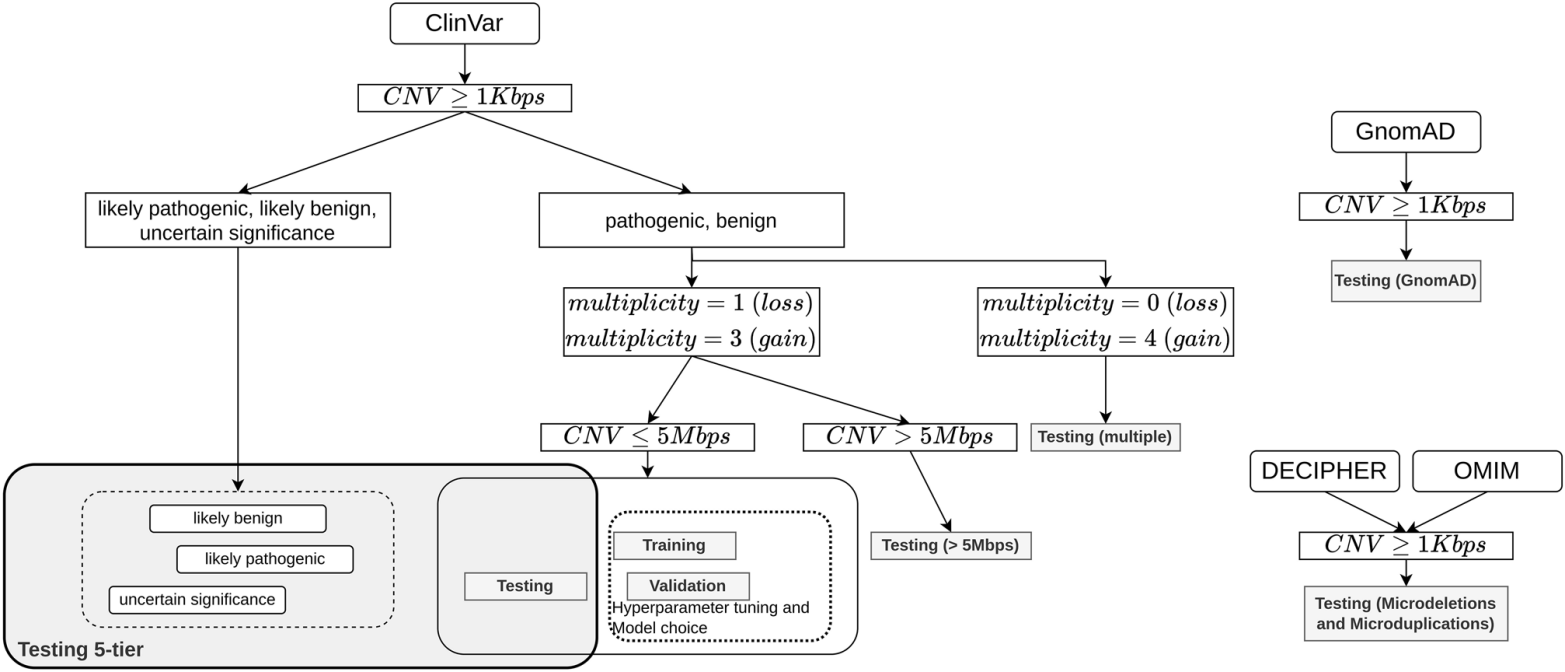
Diagram depicting used datasets and preprocessing steps. In all analyses, we only evaluated CNVs larger than 1 Kbps. CNVs with a multiplicity of 1 for losses and multiplicity of 3 for gains and smaller than 5 Mbps from ClinVar^38^ were used for training, validation of models, and basic testing for the final evaluation of the chosen model. CNVs with other multiplicity were used as an additional testing set [Testing (multiple)] as well as CNVs larger than 5 Mbps [Testing (> 5 Mbps)]. Furthermore, likely benign, likely pathogenic and CNVs of uncertain significance were also evaluated together with CNVs from the basic Testing set. Potentially benign variants were collected from the GnomAD database^39^ and pathogenic CNVs from DECIPHER^32^ and OMIM databases^22^ as additional evaluation sets (implemented with app.diagram.net^41^).

The basic steps and data sets used in the study are summarized in Fig. 1 and Table 1. Briefly, we trained the ISV method on a subset of publicly available CNV records from the ClinVar database with recorded clinical effects. We annotated them with attributes representing counts of overlapped functional elements, such as genes and regulatory elements. The annotations were then used to train a classifier to predict associated clinical classes (benign/pathogenic). The classifier was then thoroughly tested on the rest of the CNVs from the ClinVar database and on a set of presumably benign CNVs from the gnomAD population study. In addition, we tested the method on manually picked sets of established pathogenic regions from the OMIM and the DECIPHER database. A comprehensive description and preprocessing of the data is provided in the Supplementary Information in section “Datasets”. However, a compact representation of the data preparation is shown in Fig. 1.

**Table 1:** Dataset sizes after preprocessing, including only CNVs having ClinVar classification benign or pathogenic. In the case of Testing (5-tier), the labels show numbers of likely benign (+ benign) and likely pathogenic (+ pathogenic) CNVs and the “All” column contains also uncertain significance CNVs. We used the benign and pathogenic CNVs from the basic Testing set to complement the likely benign, likely pathogenic and uncertain significance CNVs.

CNV type	Dataset	Benign	Pathogenic	All
CNV gain	Training	5890	697	6587
	Validation	1256	155	1411
	Testing—basic	1261	151	1412
	Testing (> 5 Mbps)	10	1318	1328
	Testing (multiple)	382	90	472
	Testing (5-tier)	2712 + 1261	411 + 151	15,259
	Testing (GnomAD)	–	–	49,109
	Testing (microduplications)	–	33	33
CNV loss	Training	6132	2401	8533
	Validation	1292	537	1829
	Testing—basic	1289	540	1829
	Testing (> 5 Mbps)	5	1854	1859
	Testing (multiple)	2033	211	2244
	Testing (5-tier)	1806 + 1289	681 + 540	11,195
	Testing (GnomAD)	–	–	169,100
	Testing (microdeletions)	–	131	131

### Annotation of CNVs

Each CNV is annotated with features describing the counts of overlapped functional genomic elements (Supplementary Table S1). The attributes can be divided into two categories. The first category consists of gene attributes, containing the number of genes overlapped by the CNV, and their sub-categories, such as protein-coding genes, RNA elements, pseudogenes obtained from GENCODE^21^, morbid genes, and genes associated with Mendelian disease according to OMIM database^22^ (annotations gathered from AnnotSV tool^17^). Second, are regulatory elements describing counts of overlapped regulatory elements, such as promoters, promoter flanking regions, transcription factor binding sites, CTCF binding sites, enhancers, and open chromatin regions gathered from NCBI^23^.

We included counts of haploinsufficient genes and regions to copy number loss variant attributes and counts of triplosensitive regions to copy number gains attributes from the ClinGen database^24^. We only included genes and regions with haploinsufficiency/triplosensitivity scores equal to 3 (indicating that there is sufficient evidence to support a dosage sensitivity mechanism for the gene/genomic region)^15^.

The annotation was automated using the publicly available python ISV package. For reproducibility testing, we provide all of the annotated CNVs, used for training and evaluation in our study, in a table format in the project Github repository (in the “data/” folder). More information can be found in the “Code availability” and “Data availability” sections.

### Training of ISV prediction models and selection of the best performing model

For each dataset, we trained five different models for each CNV type—Linear Discriminant Analysis (LDA), Quadratic Discriminant Analysis (QDA), Logistic Regression, Random Forest implemented in scikit-learn^25^, and boosted trees (XGBoost)^26^. As the performance of each of these models depends greatly on the combination of its hyperparameters, we performed a hyperparameter grid search to find a set of hyperparameters performing best on the validation set based on Matthew's correlation coefficient^27^. The final model was chosen from the grid search results by inspecting its validation accuracy, sensitivity, specificity, and Matthew’s correlation coefficient.

### Model interpretation

To interpret the inner workings of the model, we calculated Shapley additive explanation values (SHAP)^28^. SHAP values are in theory calculated by observing the effect that each attribute contributes to the final predictions by training all possible models with and without it. As this is not feasible in practice a heuristic algorithm has to be used. The SHAP package^29^ offers easy-to-use functions for the calculation and visualization of SHAP values.

Several points need to be kept in mind when interpreting results with SHAP values. First, the concordance between attribute values and their SHAP values is not perfect, although they are usually correlated. This means that overlapping a certain number of protein-coding genes will have a different impact on the final prediction in different CNVs, influenced by the values of other predictors. This is mainly caused by the use of tree-based modeling methods.

Second, SHAP values can be negative. When fitting the explainer object, the SHAP algorithm estimates the baseline SHAP value from which all other SHAP values are added or subtracted. This can be confusing when working with probability adjustments. However, this also provides an extremely useful way of interpreting individual results by visualizing the SHAP values for individual CNVs.

## Results

### Data overview

**Figure 2:**
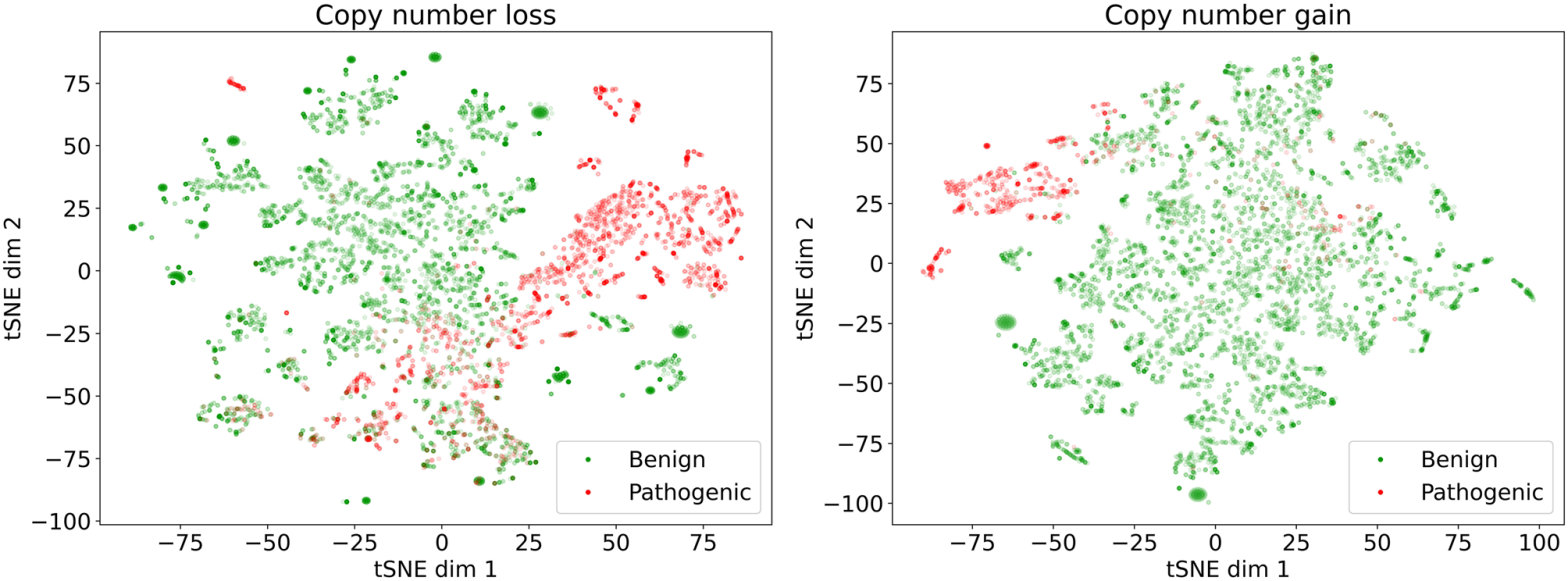
A 2-dimensional representation of the training datasets. We used the tSNE algorithm implemented in the scikit-learn package^25^ with default hyperparameters. Each dot represents a CNV, either benign (green) or pathogenic (red) (implemented with matplotlib package^42^, version 3.3.2).

We trained a model separately for copy number loss variants (8533 CNVs) and copy number gain variants (6587 CNVs) on attributes describing counts of overlapped genomic elements. During training, the ClinVar classification was considered for ground truth and each variant was labeled as either pathogenic or benign, according to its ClinVar classification. Basic descriptive analysis of the data (training and validation) is provided in Table 2. Furthermore, in the majority of the used genomic region attributes, or features (gene-related attributes and regulatory elements described in “Materials and methods”), we observed significant correlations with the clinical effect of CNV (pathogenic/benign) or both CNV types (see Supplementary Fig. S1, Supplementary Fig. S2). In Fig. 2, we provide a low dimensional data representation by the first two t-distributed Stochastic Neighbor Embedding (tSNE) components^31^. The points representing benign (green) and pathogenic (red) CNVs tend to be similar and thus closer in the attribute space. Based on this, we assume that a good classifier might exist with the selected data and attributes.

**Table 2:** Description of attributes used for training separately for copy number loss and gain variants. The aggregations [“mean” and “standard deviation” (std)] are calculated for benign and pathogenic variants separately.

Attribute	CNV loss				CNV gain			
	Benign		Pathogenic		Benign		Pathogenic	
	Mean	Std	Mean	Std	Mean	Std	Mean	Std
Overlapped gencode elements	4.11	10.49	56.43	54.73	9.46	18.39	80.3	65.18
Protein coding genes	1.41	3.29	19.28	19.49	2.61	4.6	28.17	25.22
Pseudogenes	1.42	5.46	11.3	15.62	3.97	10.85	17.34	19.58
Micro RNA	0.07	0.45	02.08	2.98	0.23	1.18	2.91	3.38
Long non-coding RNA	0.79	1.78	16.45	16.34	1.77	3.16	21.7	18.29
Ribosomal RNA	0.0	0.2	0.02	0.15	0.0	0.05	0.03	0.2
Small nuclear RNA	0.08	0.45	1.2	2.17	0.19	0.81	1.94	2.9
Morbid genes	0.2	0.48	3.84	3.65	0.37	0.7	5.22	4.6
Disease associated genes	0.18	0.46	3.24	3.11	0.34	0.68	4.26	3.85
Haploinsufficient genes	0.02	0.14	0.48	0.69	–	–	–	–
Haploinsufficient regions	0.06	0.27	0.47	0.67	–	–	–	–
Regulatory elements	18.13	40.77	453.31	396.98	40.36	59.97	559.55	424.5
Enhancers	3.35	7.55	75.64	72.11	6.7	11.01	82.97	65.57
Open chromatin regions	03.02	6.88	58.18	53.91	6.43	10.76	66.38	51.71
Promoters	1.16	3.43	35.69	37.78	2.88	5.3	51.44	49.52
Promoter flanking regions	3.41	8.43	99.24	92.16	7.72	12.95	118.6	91.8
CTCF binding sites	5.95	15.4	153.4	146.64	13.77	23.12	197.61	168.01
TF binding sites	1.11	3.38	26.93	35.99	2.6	5.47	36.9	44.69
Manually curated regulatory elements	0.12	0.61	4.25	4.66	0.26	0.73	5.64	5.28
Triplosensitive regions	–	–	–	–	0.02	0.17	0.62	0.74

### Prediction of pathogenicity of CNVs

**Figure 3:**
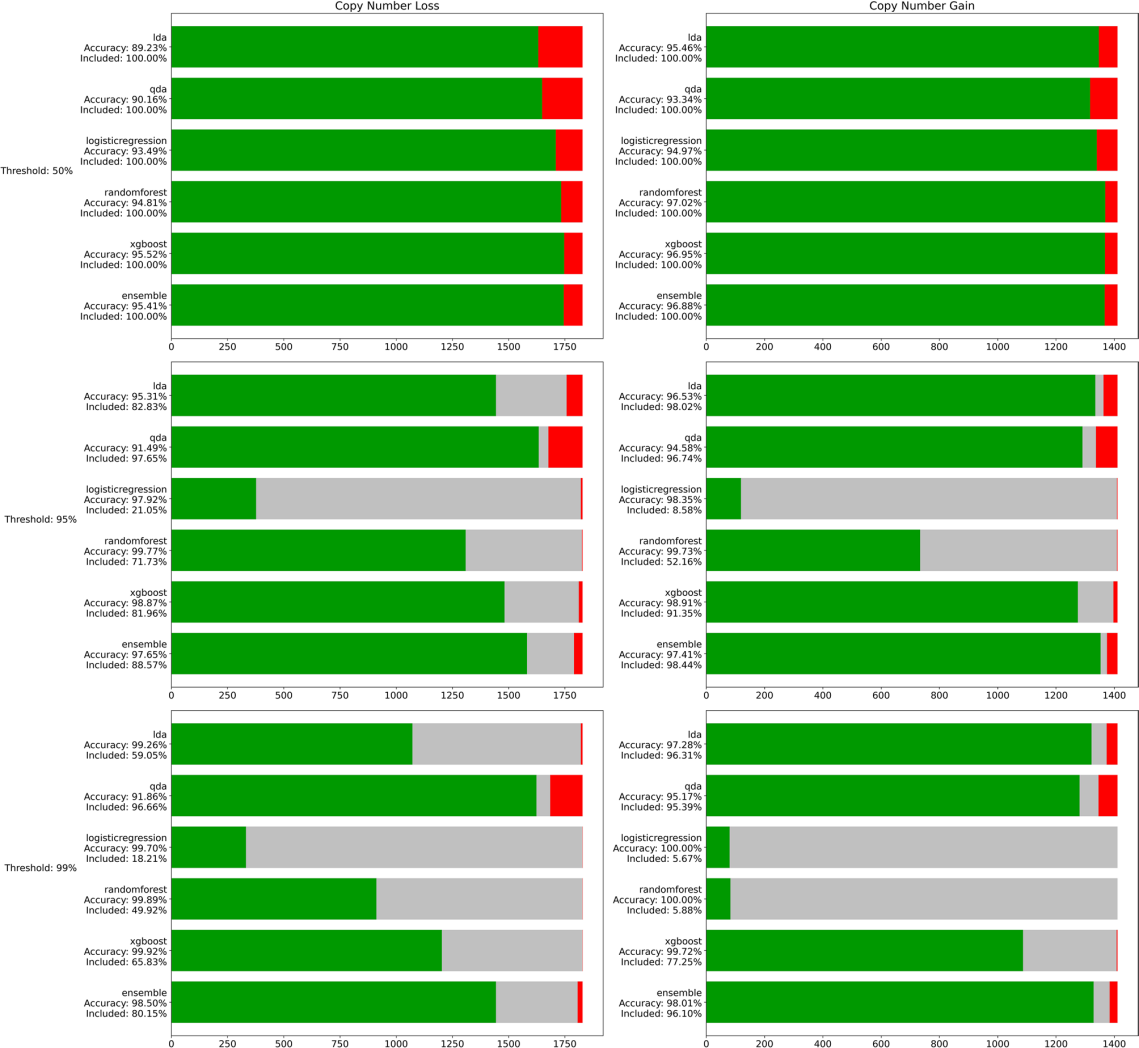
Comparison of the predictive capability of five studied models at three different probability thresholds (validation dataset). In the top row, the models classify all CNVs as either benign or pathogenic. “Correctly” predicted CNVs (being in line with ClinVar classification; either benign or pathogenic) are in green, while “incorrectly” predicted ones (that means the prediction unmatching the ClinVar classification) are in red. The middle row and the bottom row allow for uncertain predictions (shown in gray) if the probability of pathogenicity is between (1 − P_ct_, P_ct_), where P_ct_ is the probability threshold. The *x*-axis represents individual CNVs and corresponds to the sizes of the validation datasets. “Included” represents the percentage of CNVs evaluated by ISV with a clear outcome (with probabilities either above the probability threshold (P_ct_) or below 1 − P_ct_ (implemented with matplotlib package^42^, version 3.3.2 and pandas package^43^, version 1.1.3).

Since our results on the training data set suggested certain discriminatory potential we trained and compared several widely used machine-learning methods. The hyperparameter tuning and model choice was based on performance (accuracy, sensitivity, specificity, and Matthews correlation coefficient) on the validation dataset, which consisted of CNVs unseen during training. The models were trained for 100 iterations, however with early stopping set at 15 iterations. The final hyperparameters for both models are: max_depth = 8, eta (learning rate) = 0.3, gamma = 1, subsample = 1, lambda = 0.1, colsample_bytree = 0.8 and scale_pos_weight = sqrt [sum (benign CNVs)/sum (pathogenic CNVs)]. Predictions of our algorithm for these CNVs were compared to ClinVar classifications which were, again, considered as true classifications. Figure 3 depicts the comparison of five studied prediction models and their performance on validation datasets. As the models return a probability of pathogenicity (the output of tree-based methods is actually a weighted “vote”, but we will assume it as an approximation of probability further on), rather than a single discrete class representation, we allow “uncertain” predictions. Inspired by the ACMG evaluation thresholds, we evaluated our model’s performance at three different pathogenicity thresholds: P_ct_ = {0.5, 0.95, 0.99}, classifying CNVs with probability P ≥ P_ct_ as pathogenic, CNVs with P ≤ 1 − P_ct_ as benign and the rest as uncertain significance. We found the combination of the XGBoost model and threshold 0.95 the most sensible, reaching high accuracy values as well as not being too restrictive and including the majority of CNVs. However, this value can be tweaked to the user's preference and application, for example, to minimize incorrect predictions at the expense of the total yield of CNVs having a pathogenic or benign prediction.

With this model choice, to which we will further refer as ISV, the copy number loss model discovered 82.39% of benign CNVs and 76.48% of pathogenic CNVs, with 98.97% and 98.57% precision for benign and pathogenic CNVs respectively (in the testing-basic dataset). Disregarding CNVs classified as uncertain significance (representing 18.43% of CNVs), the model reached 98.86% test accuracy, 97.41% sensitivity, 99.44% specificity, 0.9719 Matthews correlation coefficient and 0.984 ROC–AUC score. Copy number gain model discovered 92.15% of benign CNVs and 76.16% of pathogenic CNVs, with 98.89% and 98.29% precision for benign and pathogenic CNVs respectively (in the testing-basic dataset). Comparison of ISV predictions against ClinVar classification is provided in Table 3. Disregarding CNVs classified as uncertain significance (representing 8.5% CNVs), the model reached 98.84% accuracy, 89.84% sensitivity, 99.83% specificity, 0.9335 Matthews correlation coefficient and 0.948 ROC–AUC score. Accuracy metrics for various thresholds are presented in Supplementary Table S2.

**Table 3:** Comparison of ISV predictions against ClinVar Classification.

CNV type	Clinvar classification	ISV—benign	ISV—pathogenic	ISV—uncertain significance
CNV loss	Benign	1062	6	221
CNV loss	Pathogenic	11	413	116
CNV gain	Benign	1162	2	97
CNV gain	Pathogenic	13	115	23

### Importance of individual genomic features

To estimate the importance of individual features on the final prediction, we fitted a SHAP explainer object to the training data using the ISV model and transformed the validation dataset. For pathogenic CNVs, the SHAP values should be large and positive, while benign CNVs should ideally have large negative SHAP values. Calculating the mean of absolute values of SHAP values for each attribute thus gives us an estimate of feature importance. The number of morbid genes turned out to be one of the most important attributes together with regulatory elements and enhancers in both gains and losses. As expected, the number of haploinsufficient genes is high on the list for losses as is the number of overlapped triplosensitive regions for gains (Supplementary Figs. S3, S4). These findings correlate well with the calculated point-biserial correlation coefficient of individual attributes in the training set (Supplementary Fig. S2).

### Evaluation of long CNVs (> 5 Mbps)

Since most of the CNVs longer than 5 Mbps from the ClinVar database were classified as pathogenic (99.2% for gains, 99.7% for losses), to prevent unwanted distortion of results, we filtered out CNVs belonging to this range and used the rest as an additional testing set [Testing (> 5 Mbp)]. The model failed to correctly predict all five long benign copy number loss variants when compared to ClinVar classification. However, this is understandable, since the model relies on raw counts of genomic elements. All of these CNVs overlapped at least 80 genes and 723 regulatory elements. As for the copy number gains, the model incorrectly predicted six out of 10 long benign CNVs and three out of 1318 pathogenic ones. In all benign cases the CNVs were overlapping at least 29 protein coding genes and at least 1234 regulatory elements. Predictions, as well as annotations, can be viewed in Supplementary Table S4. It should be noted, however, that CNVs involving genomic regions over 5 Mbps have benign clinical impact only very rarely. In our ClinVar derived data set they represented 0.8% among gains and 0.3% among loss CNVs (Testing (> 5 Mbps); Table 1).

### Evaluation of CNV multiplicity

We evaluated the model on CNVs deleted on both copies of chromosomes (i.e. multiplicity = 0) in case of losses, or CNVs amplified twice (i.e. multiplicity = 4) for copy number gains. On copy number losses the model reached 98.81% accuracy, 82.5% sensitivity, and 99.59% specificity while interpreting 21.21% CNVs as uncertain significance. On copy number gains the model reached 99.56% accuracy, 97.7% sensitivity, and 100% specificity (see Supplementary Table S2) while interpreting 4.66% of CNVs uncertain significance. These results are similar to test results except for slightly decreased sensitivity in copy number losses but increased sensitivity for copy number gains.

### Evaluation of likely benign, likely pathogenic, and CNVs of uncertain significance

The models were trained and evaluated only on CNVs with a clear label (benign or pathogenic) provided by the ClinVar database. However, many CNVs are yet of unknown or not fully understood significance, therefore many of them are labeled as likely benign, likely pathogenic or uncertain significance. Assuming only benign and pathogenic variants without reporting the model’s behavior on the rest of CNVs can lead to a potentially biased model since many of the CNVs, for which we are sure of their clinical significance, might be the extremes of some unknown distributions for which we are estimating the decision boundary. Therefore, we evaluated and tested the ISV model (XGBoost with threshold = 0.95) also in the context of ClinVar CNVs being classified using the whole range of the five-tier system.

**Figure 4:**
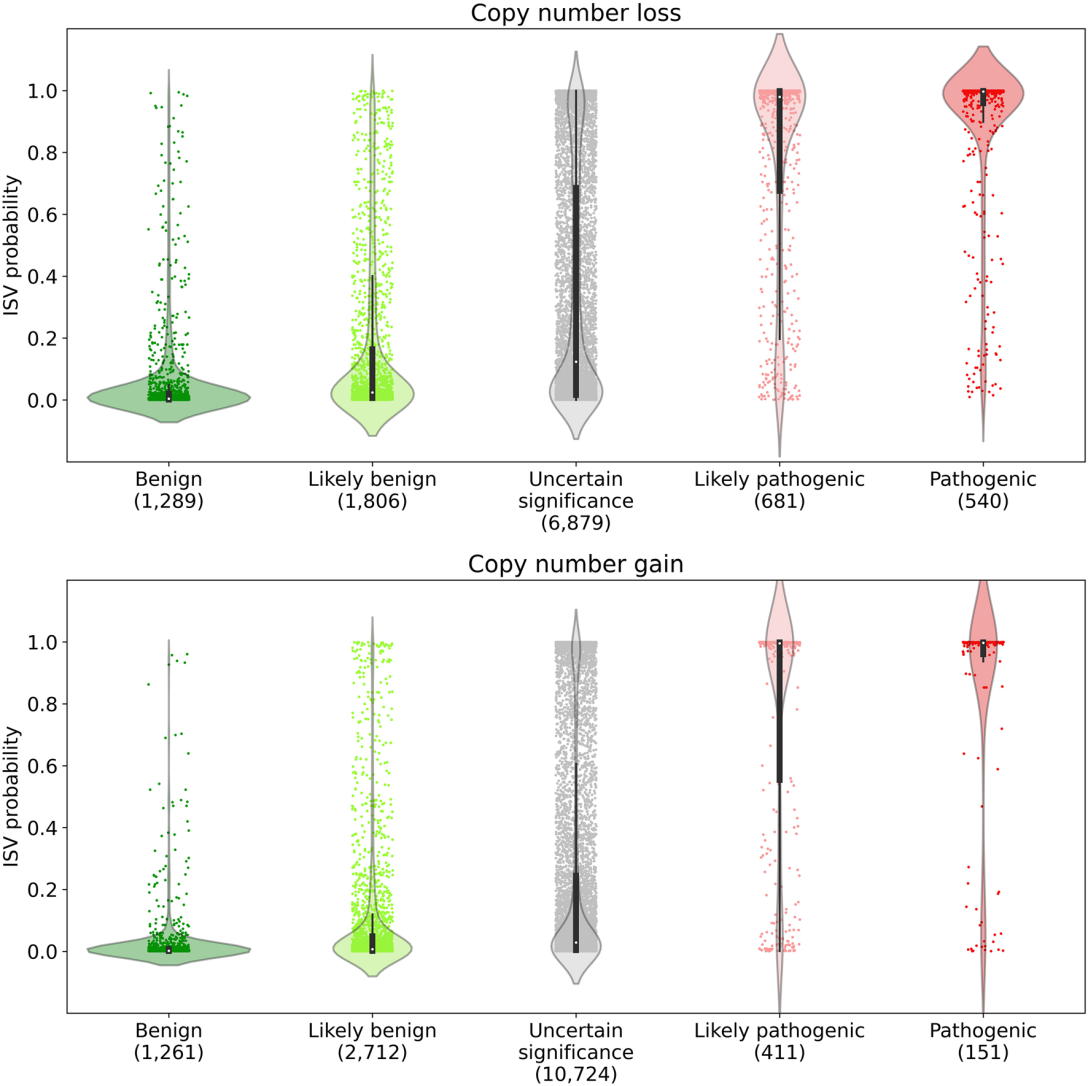
Evaluation of ISV on CNVs with standard five-tier classification generally used for the classification of genomic variants in Mendelian diseases. Each CNV is represented by a dot while the color patterns reflect purely the five-tier ClinVar classification, i.e. neither the ISV prediction nor the “matching” status between ISV and ClinVar. The ISV prediction of pathogenicity is reflected on the *y*-axis while the value 1.0 means pathogenic prediction and 0.0 means benign prediction. Please note that these classes of variants are recommended by the respective ACMG/AMP guidelines^44^. The sizes of datasets are provided in parentheses under the classification labels (implemented with seaborn package^45^, version 0.11.0).

When considering the distributions of predicted probabilities for each CNV (either copy number gain or loss) grouped according to the five classes (according to ClinVar), it is clear that the ISV model predicts the majority of ClinVar likely benign CNVs as benign and ClinVar likely pathogenic ones as pathogenic (Fig. 4). When comparing these to the benign and pathogenic groups, however, the distributions were wider in the likely benign/pathogenic groups and there were more edge cases in these categories, shown by a higher number of unmatching CNVs between ISV prediction and ClinVar classification. Moreover, CNVs with ClinVar classification of uncertain significance were distributed throughout the whole range of pathogenicity predictions. They showed, however, clear bimodal clustering at both ends of the distribution, suggesting certain potential for further improvement of classification of CNVs, for example by exploiting their potential in a semi-supervised learning scenario, which could lead to an even more robust model.

### Comparison with other methods

An existing method, ClassifyCNV^16^ for classifying CNVs based on automatic evaluation of the ACMG criteria^15^ achieves relatively high accuracy, although rather conservatively classifies the majority of CNVs as uncertain significance. According to the publication^16^, the method is able to discover 57% of all truly pathogenic/likely pathogenic CNVs and 11.8% of truly benign/likely benign. For consistency, we evaluated the list of CNVs in the test set with both ClassifyCNV and ISV to compare the performance of these methods. AnnotSV^17^ evaluates the severity of CNV according to ACMG criteria as well, so it is also included in the comparison. Finally, we also evaluated the performance of the StrVCTVRE^19^ program on the test data, which is a machine learning-based method (briefly explained in the introduction).

**Figure 5:**
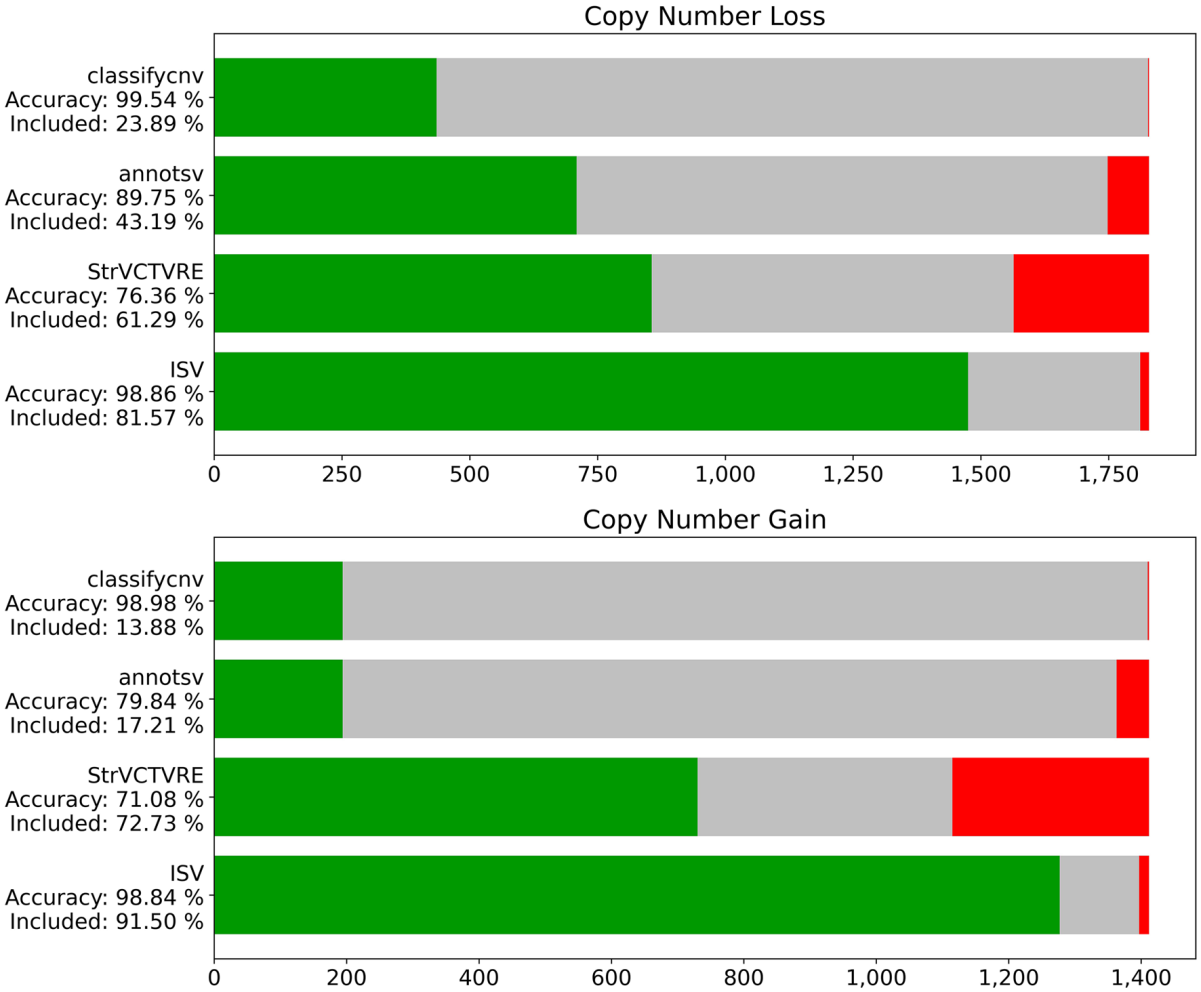
Numbers of correct (green), incorrect (red), and uncertain (gray) predictions on the test data. For ClassifyCNV and AnnotSV we treated likely benign and likely pathogenic predictions as uncertain significance. If we treated them as benign/pathogenic instead, we observed an increase in false predictions, while the added percentage of CNVs was not enough to categorize this as an improvement in the model’s performance (see Supplementary Fig. S11). The StrVCTVRE algorithm only classifies exonic CNVs, thus the ones shown as uncertain significance correspond to ones outside of exonic regions (implemented with matplotlib package^42^, version 3.3.2 and pandas package^43^, version 1.1.3).

We show in Fig. 5 that ISV was able to correctly classify most CNVs, however, at the cost of producing more incorrect predictions than ClassifyCNV which, on the other hand, resulted in a significantly higher number of uncertain predictions. However, this can be mitigated by enforcing a stricter probability threshold. The StrVCTVRE algorithm yielded the lowest accuracies of all methods, reaching 76.36% for copy number losses and 71.08% for copy number gains.

### Evaluation of GnomAD variants

**Figure 6:**
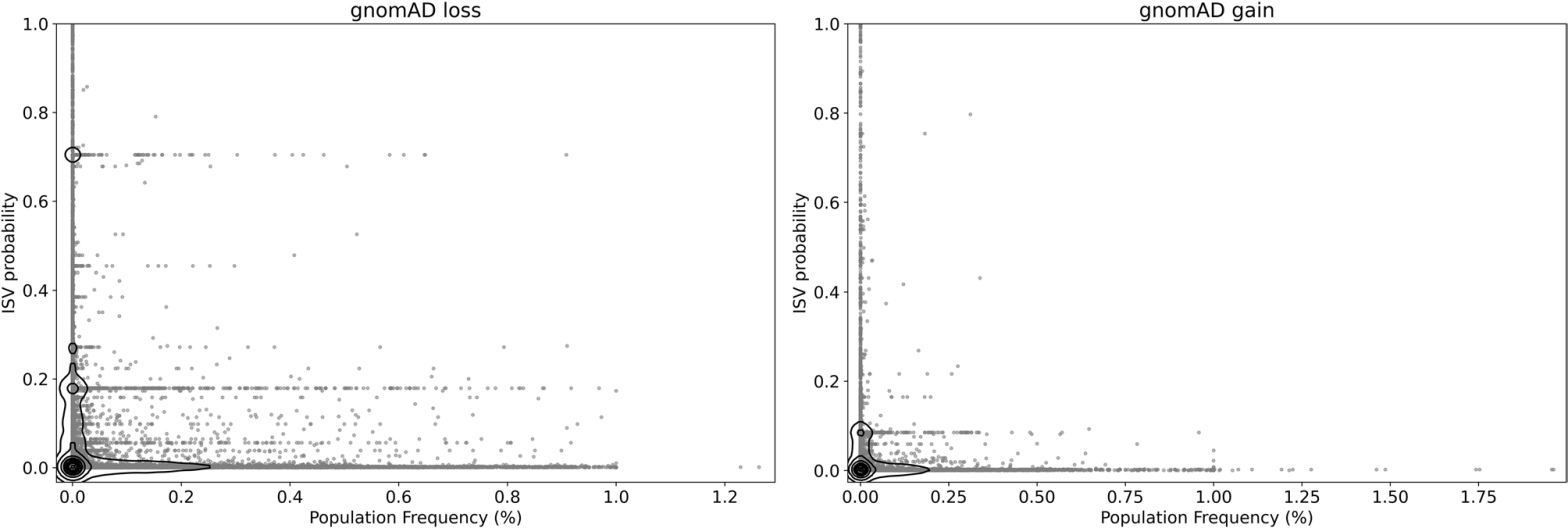
Evaluation of ISV tool on gnomAD data. The *x*-axis represents the population frequencies of CNVs (black dots) with the ISV probability of pathogenicity on the *y*-axis. The figure shows that the majority of frequently occurring CNVs were classified as benign by ISV, while the ones with a higher probability of pathogenicity occur rarely (implemented with seaborn package^45^, version 0.11.0).

The GnomAD database offers records of structural variation with extensive population-specific descriptions, as well as summarizations across the populations. Due to evolutionary pressure, we expect variants occurring at higher frequencies to be under low selective pressure, while the opposite should hold for potentially disruptive/pathogenic variants. Figure 6 shows that in both copy number loss and copy number gain we observed variants classified as pathogenic by ISV occurring at low population frequencies and variants with low pathogenic probability occurring at a wider range of frequencies. This matches our expectations where variants occurring at higher frequencies should have a lower probability of pathogenicity.

### Evaluation of pathogenic microdeletions and microduplications

**Figure 7:**
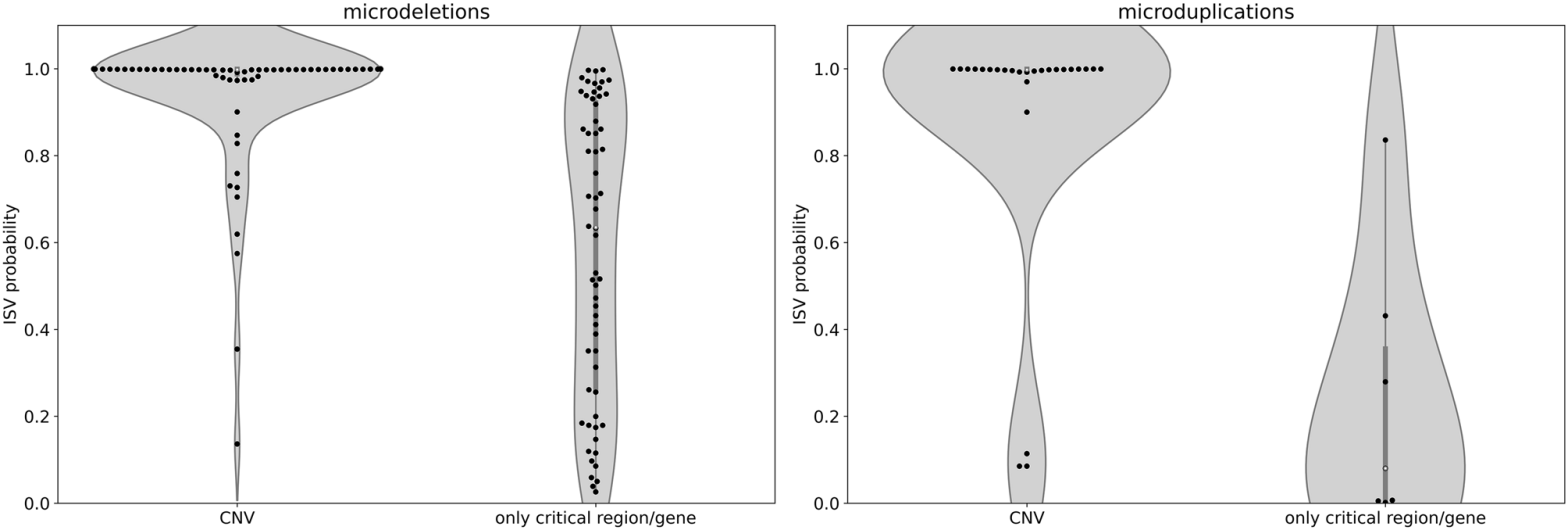
Evaluation of pathogenic microdeletions and evaluation of pathogenic microduplications is stratified into two classes, showing that inclusion of critical region/gene may not be sufficient for correct prediction (implemented with seaborn package^45^, version 0.11.0).

The ISV tool should have both high confidence for predictions of benign variants as well as known pathogenic ones. In the previous section, we showed that potentially benign variants are predicted with ISV with a low probability of pathogenicity. To showcase the performance on pathogenic CNVs we collected known microdeletion and microduplication syndromes from DECIPHER^32^ and OMIM^22^ databases. Of the 164 evaluated pathogenic microdeletions/microduplications, ISV would classify most CNVs (91) as pathogenic, five as benign and the rest (68) as uncertain significance (Supplementary Table S3). We observed that the majority of CNVs with a low probability of pathogenicity contained only the most critical region, meaning that absolute numbers of overlapped genomic elements were low. Therefore we show in Fig. 7 that knowing the coordinates of the CNV and not just the overlapped critical region/gene yields better overall predictions.

### Results interpretation and data visualisation

**Figure 8:**
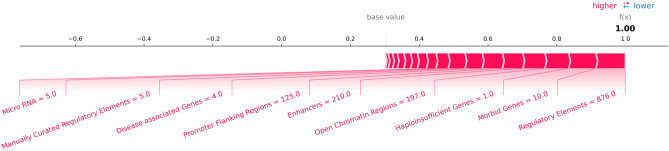
Force plot showing contributions of individual attributes towards the final prediction for a CNV causing Prader-Willi and Angelman syndrome (chr15:22760000–28560000). Bars represent individual attributes contributing to the prediction of this CNV with bar widths reflecting the strength of each attribute. In this case, all attributes contribute to the pathogenicity of the CNV, however, this will not always be the case. The base value represents the prior baseline value, from which the individual contributions are added/subtracted. If values of all attributes were equal to 0, the final prediction would be equal to the base value. Attributes are in order according to their strength in the prediction while “regulatory elements” being the most contributing genomic attribute. Hi-genes = haploinsufficient genes. The plot was constructed by utilizing functions from the SHAP package^29^ (version 0.37.0).

Explaining the inner workings of a complex model plays a crucial part right after predicting the output of a sample. Calculation of SHAP values is a great way of estimating the contribution of each attribute to the final prediction. Knowing how the value of each attribute contributed to the final prediction can be useful in (not only) difficult interpretation cases and can help clinicians to focus their effort on a particular set of attributes. We picked five well studied pathogenic copy number loss variants (from ISCA database^33^) to showcase the model interpretability using SHAP values for each genomic feature included in the prediction: DiGeorge syndrome (chr22:18660000–21520000), Prader-Willi and Angelman syndrome (chr15:22760000–28560000), Cri-du-chat syndrome (chr5:0–15680000), 1p36 deletion syndrome (chr1:560000–21600000) and Wolf-Hirschhorn syndrome (chr4:80000–2020000) (all genome coordinates correspond with the GRCh38 genome assembly). To improve user experiences and to better understand the results of each prediction on an individual CNV level, ISV allows users to visualize and evaluate the contributions of each attribute to the final prediction on a probability scale. This can be visualized in a form of a detailed waterfall plot of SHAP values (Supplementary Figs. S5–S9 for each of the above-mentioned examples), as well as in a compact version of the same waterfall plot (Fig. 8 for Prader-Willi and Angelman syndrome (force plot)) (generated by SHAP package^29^).

### Genome annotation with ISV

**Figure 9:**
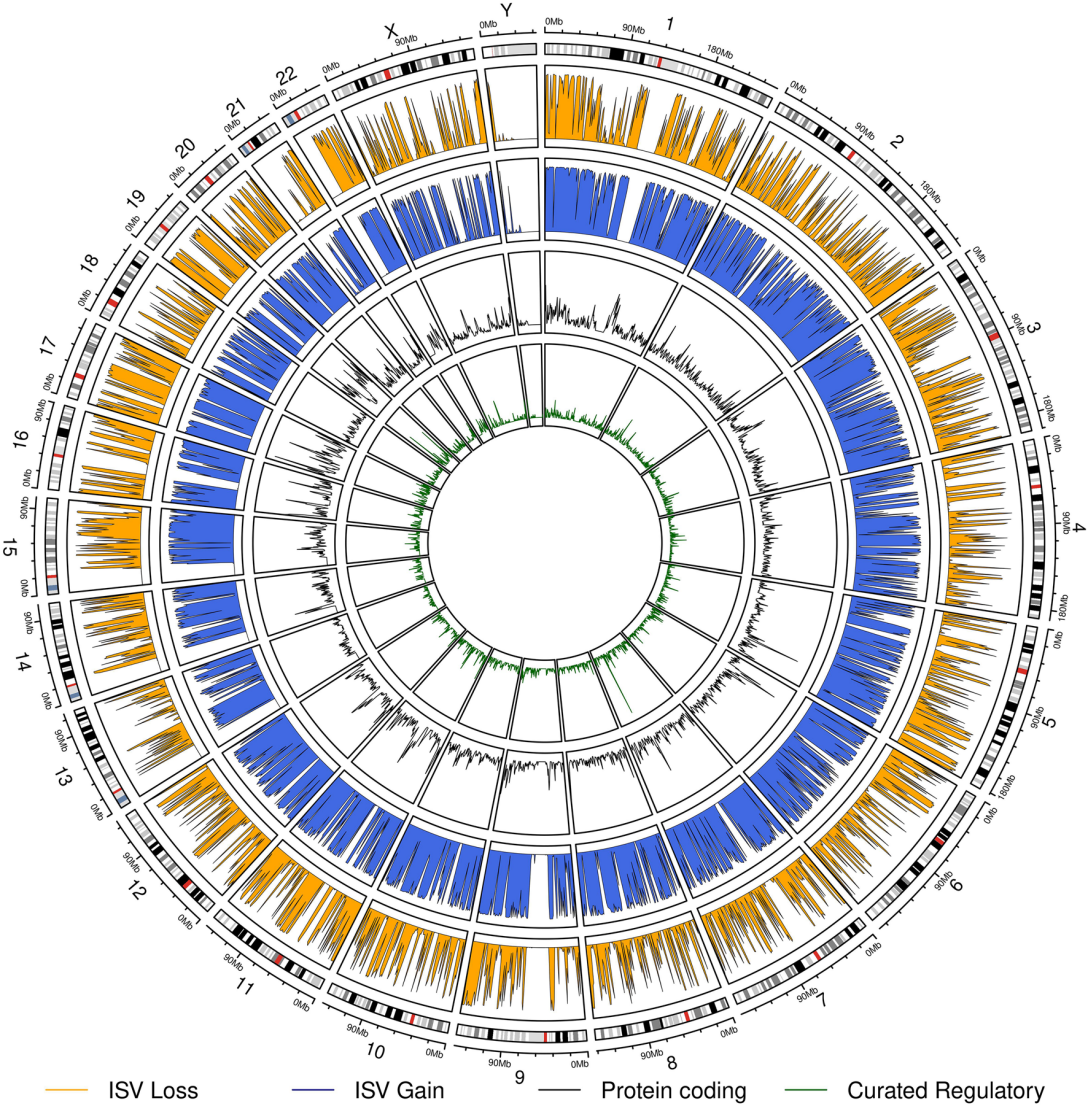
Circular genome plot with annotations by ISV. We divided the genome into 1 Mbp long non-overlapping CNVs and predicted their impact with ISV. The orange track shows probabilities of pathogenicity for copy number loss variants while the blue track shows this for copy number gain variants. The two inner tracks show the numbers of overlapped protein coding genes (black line) and overlapped curated regulatory elements (green line). The outer track shows the estimated chromosome bands according to the G-banding pattern^34^. The plot was constructed using the R package circlize^46^, version 0.4.2.

As our method relies on counts of known genes and regulatory elements and their types, we could annotate and evaluate the pathogenicity of any CNV in the genome regardless of whether it was reported in any database. We decided to split the human reference genome (GRCh38) to 1 Mbp long non-overlapping CNVs and predicted their pathogenicity with ISV. When considering the distribution of pathogenicity prediction values of ISV throughout the genome a great variability is visible for both copy number losses and copy number gains (Fig. 9).

The outer track shows the G-banding pattern, where the dark (G-positive) bands tend to be heterochromatic and AT-rich, while the bright regions are mostly euchromatic and rich for GC pairs^34^. Since GC content is strongly correlated with biological features of genome organization, such as gene density^35^, brighter bands may be more prone to pathogenic effects of CNV. As expected the ISV predictions in the regions were in line with the functional and nucleotide content of the affected regions. We observed elevated prediction of pathogenicity in active euchromatic regions (Fig. 9, Outer track, Bright regions; Supplementary Fig. S10) compared to the heterochromatic regions (Fig. 9, Outer track, Dark regions; Supplementary Fig. S10) according to G-banding pattern^34^. Also, differing predictions along the genome further confirmed our assumption that the length alone is not a sufficient predictor of the CNV pathogenicity (Supplementary Section “Association of CNV length with pathogenicity”).

## Discussion

Although CNVs belong to those genetic variations which were described among the first ones, specifically in connection to human pathologies, improving molecular genetic methods at the beginning of the twenty-first century led to an increased interest in them and thus also to an exponential increase in knowledge about their biomedical relevance^2^. It became evident that CNVs are relatively common in human populations and that assessment of their clinical importance may be challenging, especially in those which are not so large as to be unambiguously pathogenic. Several tools have been proposed for CNVs characterization, annotation, or even interpretation^1^ such as SVscore^14^ which predicts pathogenicity of CNVs by aggregating per base SNP pathogenicity scores. A more recent tool StrVCTVRE^19^ is a machine learning-based tool that evaluates exomic CNVs based on attributes describing gene importance, coding regions, conservation, expression, and exon structure. Each tool provides specific information contributing to CNVs interpretation and a better understanding of the functional impact of such variants, however, they also have various limitations. In the clinical or research setting, therefore, it is valuable to aggregate information from multiple such tools for accurate interpretation of analyzed CNVs. Moreover, CNV prediction programs have shown high uninformative counts, requiring additional manual entry from the users, which may be considered as one of the major limiting factors for the applicability of these programs in clinical applications^36^. Still, the biggest limitation concerns the final classification of variants according to their most likely clinical significance. To ease manual ACMG classification into five classes, there are, however, also tools which were designed to either manually set individual ACMG criteria^15^, such as ClinGen CNV Pathogenicity Calculator^37^, or to facilitate automated classification based on an automated selection of met criteria, such as ClassifyCNV^16^.

For the above-mentioned shortcomings of CNV prediction algorithms, we aimed to design and create an automated method encompassing various parameters in order to predict the most likely clinical significance of individual CNVs. The method requires only basic information about the position and type of a CNV, i.e. genomic coordinates and whether there is a loss or gain of the genomic region. The CNV is then annotated using different databases, with attributes describing counts of gene and regulatory elements involved in the CNV region, which are subsequently evaluated by the trained model called ISV. Based on these elements, ISV predicts the likely clinical impact of CNVs that may fall into the benign or pathogenic categories. In addition to the two basic categories, we advise, however, using a more conservative model (such as we did in the “Results” section) to allow for predictions of uncertain significance too, with probabilities between artificially chosen threshold values. In our case the 0.05 and 0.95 thresholds worked well, however, these numbers can be tweaked to the user’s preference and the requirements of the application. Final classification of most likely clinical significance, for example using a generally accepted five-tier system^15^, is not included among the features of ISV and will need to rely on other tools or manual classification of clinical or laboratory experts.

For a graphical interpretation of a model's behavior, we strongly recommend computing and plotting SHAP^29^ explanation values. The scripts from the SHAP package are very easy to use, however with limited customization. In our project repository, we offer a custom waterfall plot function (at “./scripts/plots/waterfall.py”), which can be extended freely to the user’s preference. The waterfall plots show contributions of attributes to the final predictions, uncovering the inner workings of the model. Looking at the SHAP values can help even in cases when the pathogenic potential of a CNV is not clearly defined, by providing a useful summary and direction for the researcher seeking to discern intricate copy number variants.

We have shown that the numbers of overlapped genomic elements can be used to estimate CNV pathogenicity with high accuracy (~ 98%). In most cases ISV will produce sensible predictions as we have proven on evaluations on ClinVar derived data^38^, gnomAD data^39^, and also on known manually collected pathogenic microdeletions and microduplication from OMIM^22^ and Decipher databases^32^. On the other hand, although ISV works reasonably well in general, we provide several cases of CNVs where ISV failed to provide expected predictions, with a thorough look into each CNV (Supplementary Discussion section of Supplementary Information). With this regard, it should be noted that during our analyses we uncovered several shortcomings of ISV-based predictions.

There are at least two main shortcomings of our tool, which should be mentioned explicitly. One of these is the uninformativeness about the individual genomic elements, i.e., that ISV does not inform about the impact of individual genes or genomic elements, rather it gives information about an overall effect of each element type on the prediction. Other limitations arise from the fact that ISV uses counts of overlapped genomic elements only. Specifically, the evaluation of CNVs affecting a relatively small number of genes and regulatory regions could represent challenges for ISV predictions, for example, if these overlapped genomic elements are critical for pathogenic predictions. This may happen in cases if the presence or absence of a single gene determines whether a variant will be pathogenic or benign). In addition to not considering the severity and importance of individual overlapped genes and genomic elements, a possible network of associations between individual elements is also disregarded by ISV, so the overall accuracy could be further improved in the future, but a completely different solution will be required (e.g. a model paying attention to each overlapped element, rather than aggregating the information in terms of counts). However, it should also be noted that the resolution of the CNV detection, i.e. the bin size, is the important factor that should be considered, as it could affect the result of prediction. In general, with increasing bin size, we expect larger deviations from the true CNV breakpoints. This applies especially at the loci where a morbid gene or essential genomic region contributing to pathogenicity is located a few kb upstream or downstream of predicted CNV breakpoints.

Although ISV represents an in silico prediction tool with higher than previously reported performance, including a lower percentage of CNVs with uncertain significance prediction result, there are several of the above-mentioned limitations which are still present and with which it is necessary to deal in the future or at least keep them in mind when evaluating the results of ISV predictions. Although there is a significantly higher amount of correct predictions (depending on the exact parameters used) resulting in a lower number of uninformative cases, false results are still present (again, depending on the exact parameters used). Therefore, it is inevitable to understand that ISV is only a prediction tool and thus, manual curation of the results is still necessary, especially before using them in the clinical decision-making process. Therefore, we recommend pairing the predictions up with another method or with stringent classification using well-defined standards, such as the ACMG criteria for variant classification, which will pay more attention to individual critical overlapped elements (such as haploinsufficient genes) and other specific circumstances relevant to individual CNVs and clinical cases. On the other hand, although being based on machine learning algorithms, ISV comes with an intuitive and understandable graphical interface to communicate the attributes which contributed to the prediction, together with their effect, certainly facilitating this necessary oversight. We believe that the method can be improved in the future, as many genomic databases are expanding and new CNVs are being annotated. Furthermore, we assume that a predictor utilizing more detailed features of affected elements, such as gene annotations representing their conservancy and known clinical impact^40^ should make the decision process even more precise.

## Supplementary Information

Supplementary Information.

Supplementary Table S1.

Supplementary Table S2.

Supplementary Table S3.

Supplementary Table S4.

## Data Availability

Trained models together with all the datasets can be accessed at: https://github.com/tsladecek/isv_cnv.

## Code availability

The entire project pipeline is written in snakemake^30^. The results can be exactly reproduced by following instructions at https://github.com/tsladecek/isv_cnv. We also offer a command line tool for easy and fast annotation and prediction of pathogenicity of CNVs at https://github.com/tsladecek/isv_package. It is also available as a pip package at https://pypi.org/project/isv/.
