# Supplementary material for: Automated prediction of the clinical impact of structural copy number variations
Source: Sci Rep. 2022 Jan 11;12:555. doi: 10.1038/s41598-021-04505-z (PMC8752772; doi:10.1038/s41598-021-04505-z)
Supplement: Supplementary file 1 — Supplementary Information. [file 41598_2021_4505_MOESM1_ESM.pdf]

Supplementary Information

Automated prediction of the clinical impact of structural copy number variations

Gažiová M.[1,2,+], Sládeček T.[1,+], Pös O.[1,3, +], Števko M.[1], Krampl W.[1,3,5], Pös Z.[1,3,4], Hekel R.[1,5,6], Hlavačka M.[1], Kucharík M.[1,5], Radvánszky J.[1,4,5], Budiš J.[1,5,6*], Szemes T.[1,3,5]

[1] Geneton s.r.o., Bratislava 84104, Slovakia
[2] Department of Computer Science, Faculty of Mathematics, Physics and Informatics, Comenius University, Bratislava 84248, Slovakia
[3] Department of Molecular Biology, Faculty of Natural Sciences, Comenius University, Bratislava 84215, Slovakia
[4] Institute of Clinical and Translational Research, Biomedical Research Center, Slovak Academy of Sciences, Bratislava 84505, Slovakia
[5] Comenius University Science Park, Bratislava 84104, Slovakia
[6] Slovak Center of Scientific and Technical Information, Bratislava 81104, Slovakia

* Corresponding author: Jaroslav Budis jaroslav.budis@geneton.sk

+ These authors contributed equally to this work

Supplementary Figures

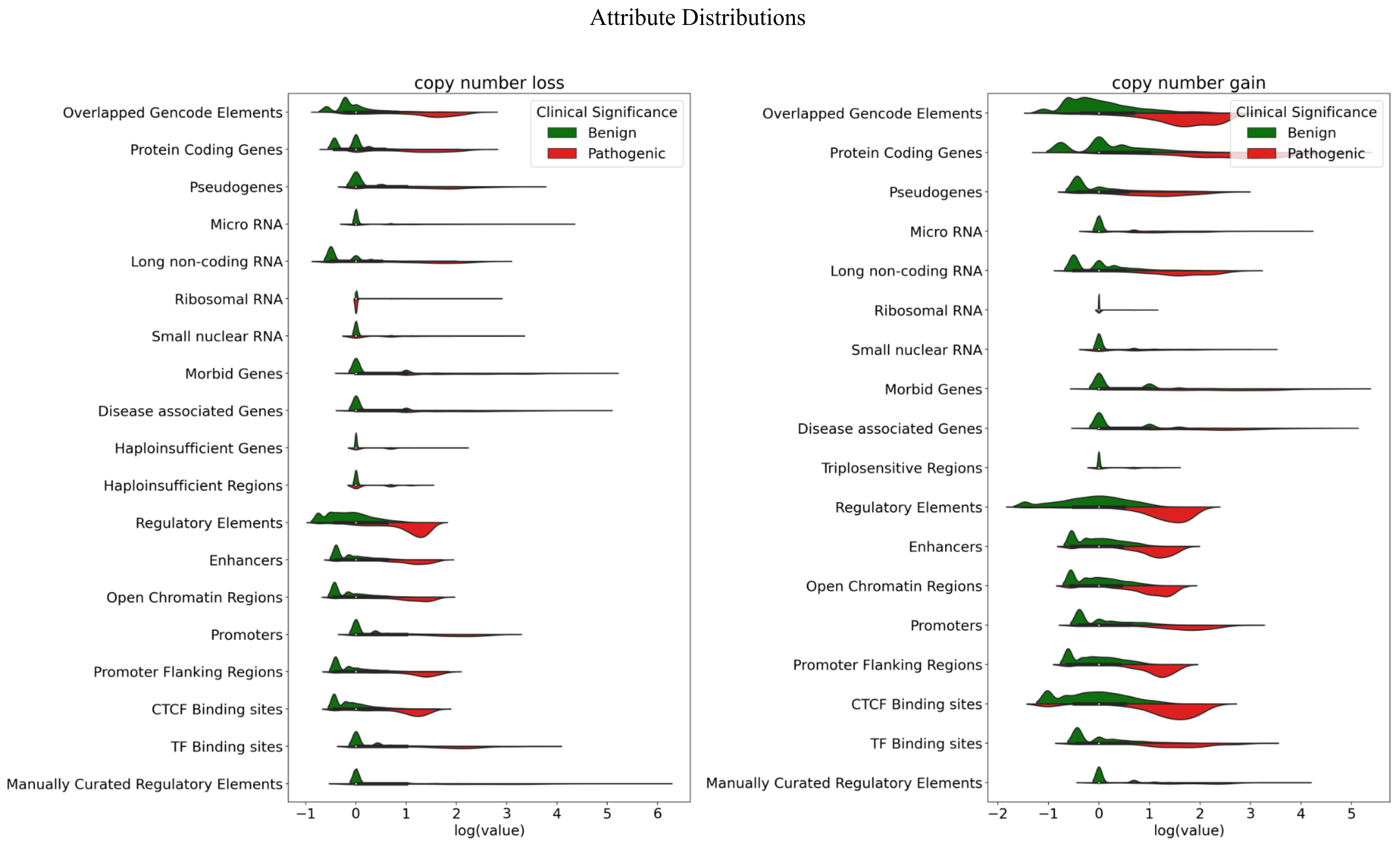

Supplementary Figure S1: Distributions of counts of overlapped genomic elements on a logarithmic (natural) scale

Correlations with the label (benign or pathogenic)

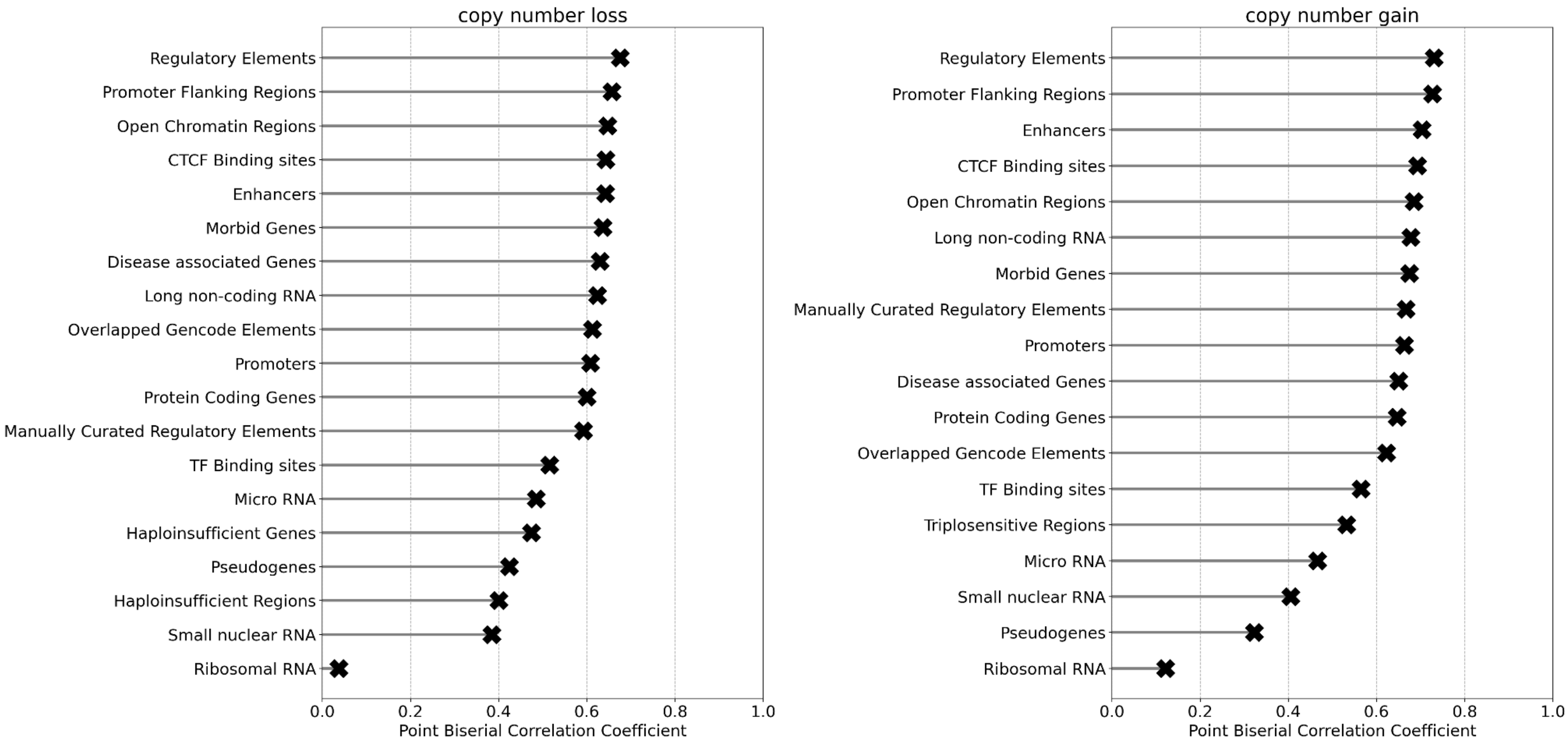

Supplementary Figure S2: Point-Biserial Correlation Coefficient of individual attributes with the label (benign or pathogenic)

Distribution of SHAP values for Copy Number Gain variants

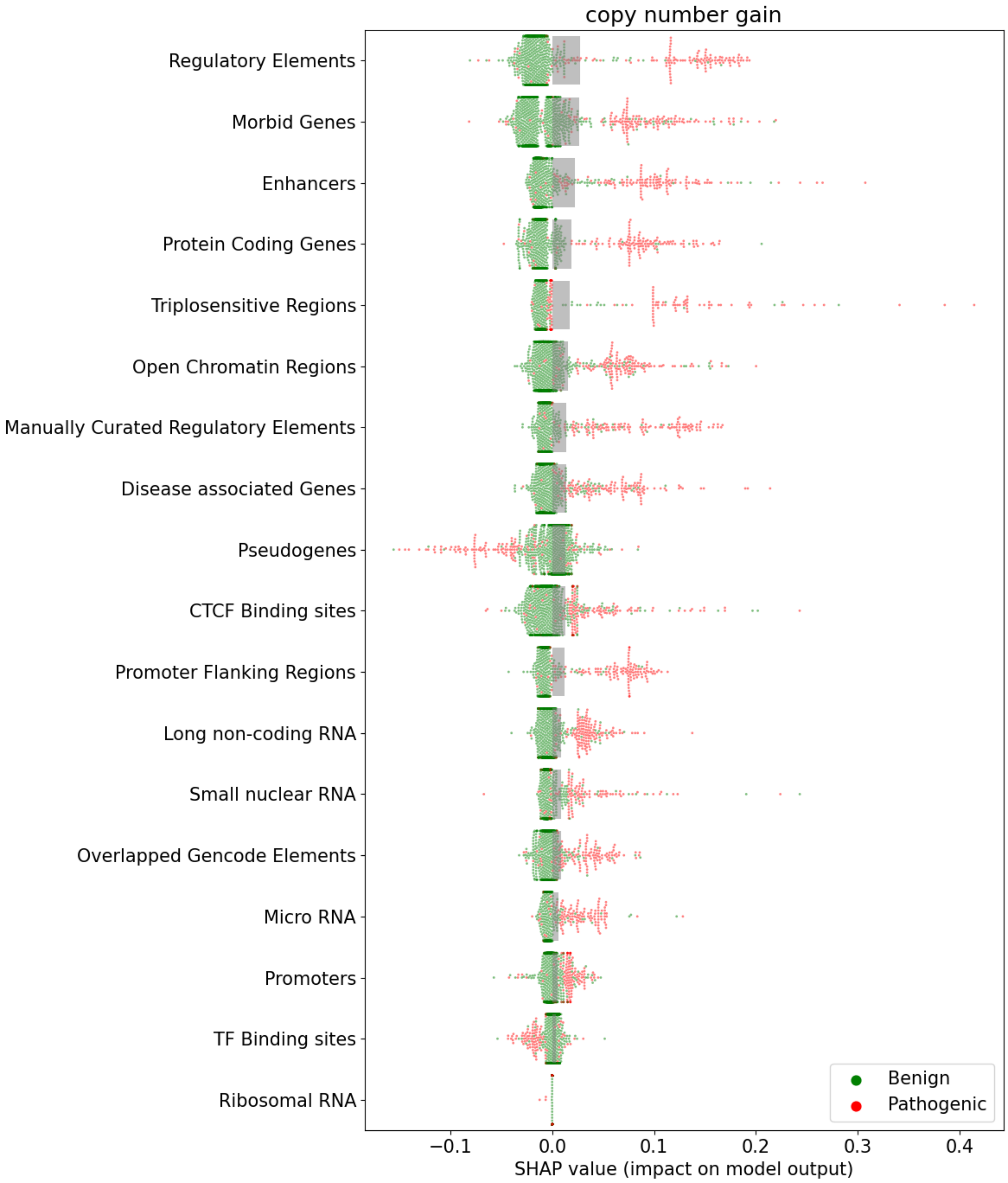

Supplementary Figure S3: Distribution of SHAP values (probabilities) for CNVs in a validation set of copy number gain variants. Attributes are sorted by the mean of absolute values of SHAP values, which corresponds to their predictive importance. Ideally, pathogenic CNVs should have high SHAP values, while benign CNVs should have lower SHAP values.

Distribution of SHAP values for Copy Number Loss variants

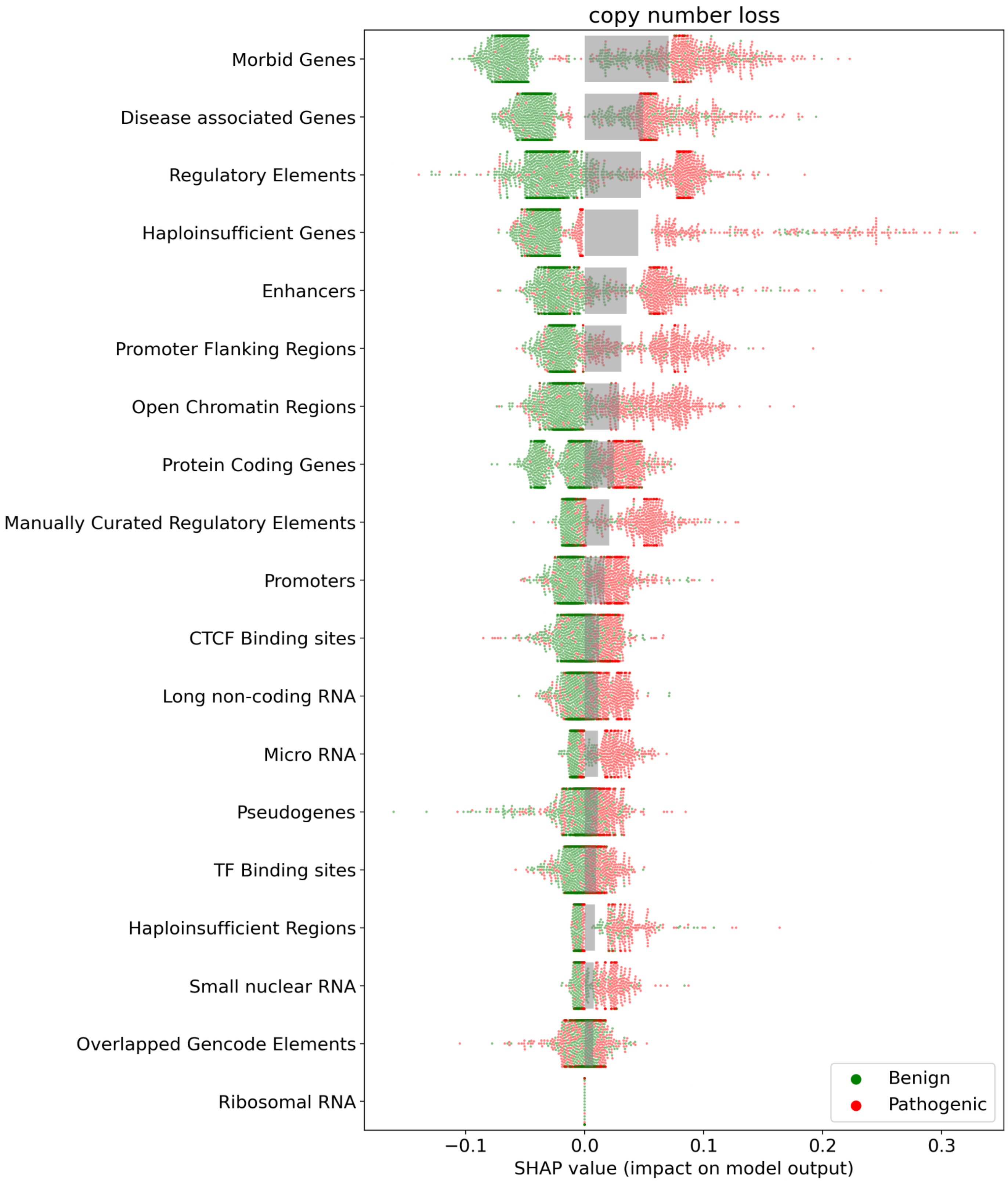

Supplementary Figure S4: Distribution of SHAP values (probabilities) for CNVs in a validation set of copy number loss variants. Attributes are sorted by the mean of absolute values of SHAP values, which corresponds to their predictive importance. Ideally, pathogenic CNVs should have high SHAP values, while benign CNVs should have lower SHAP values.

Model Interpretation with SHAP values - DiGeorge syndrome

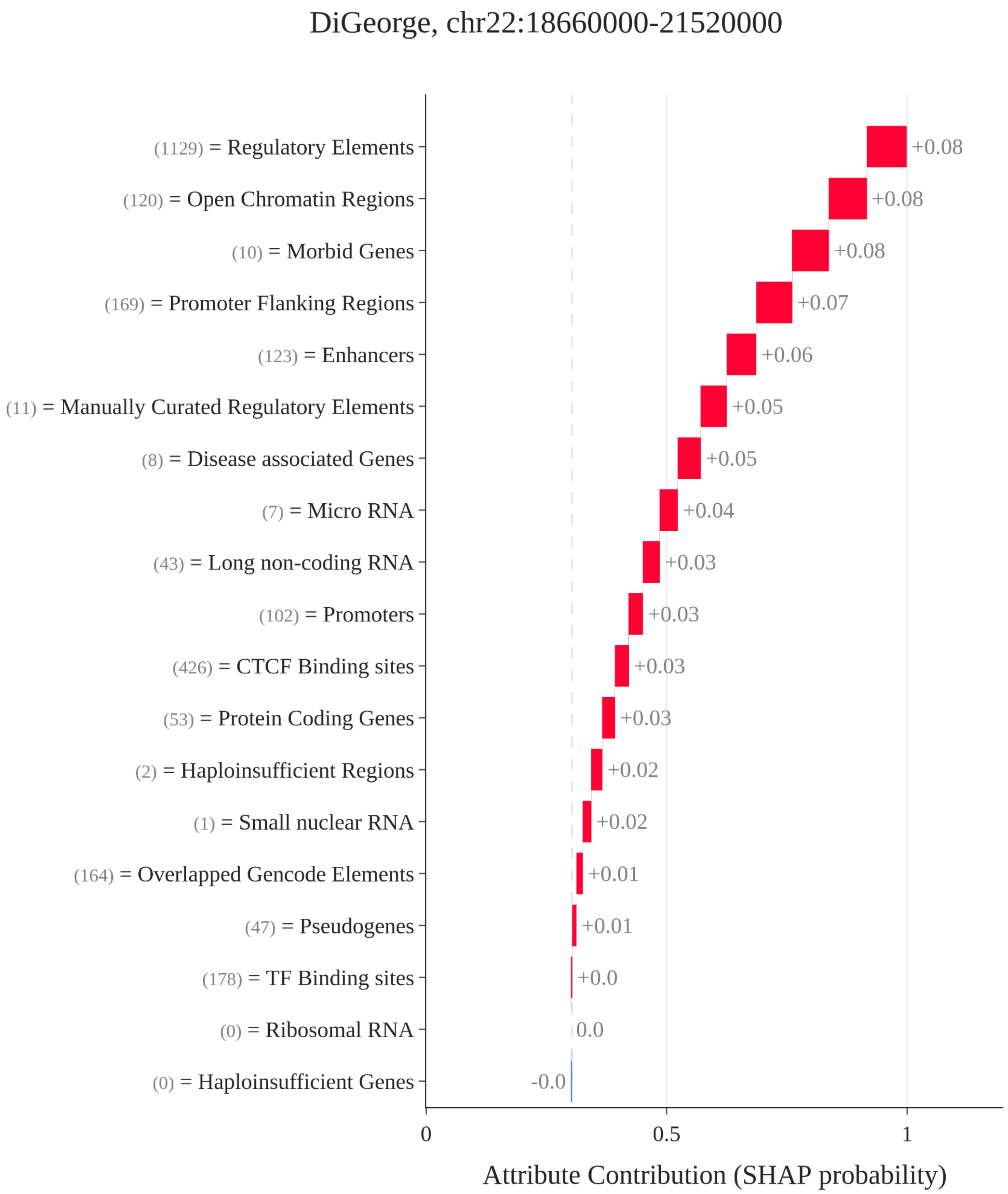

Supplementary Figure S5: Waterfall plot of SHAP value effects of each attribute on the final prediction for a variant responsible for the DiGeorge syndrome. The attributes are sorted by the absolute value of contribution.

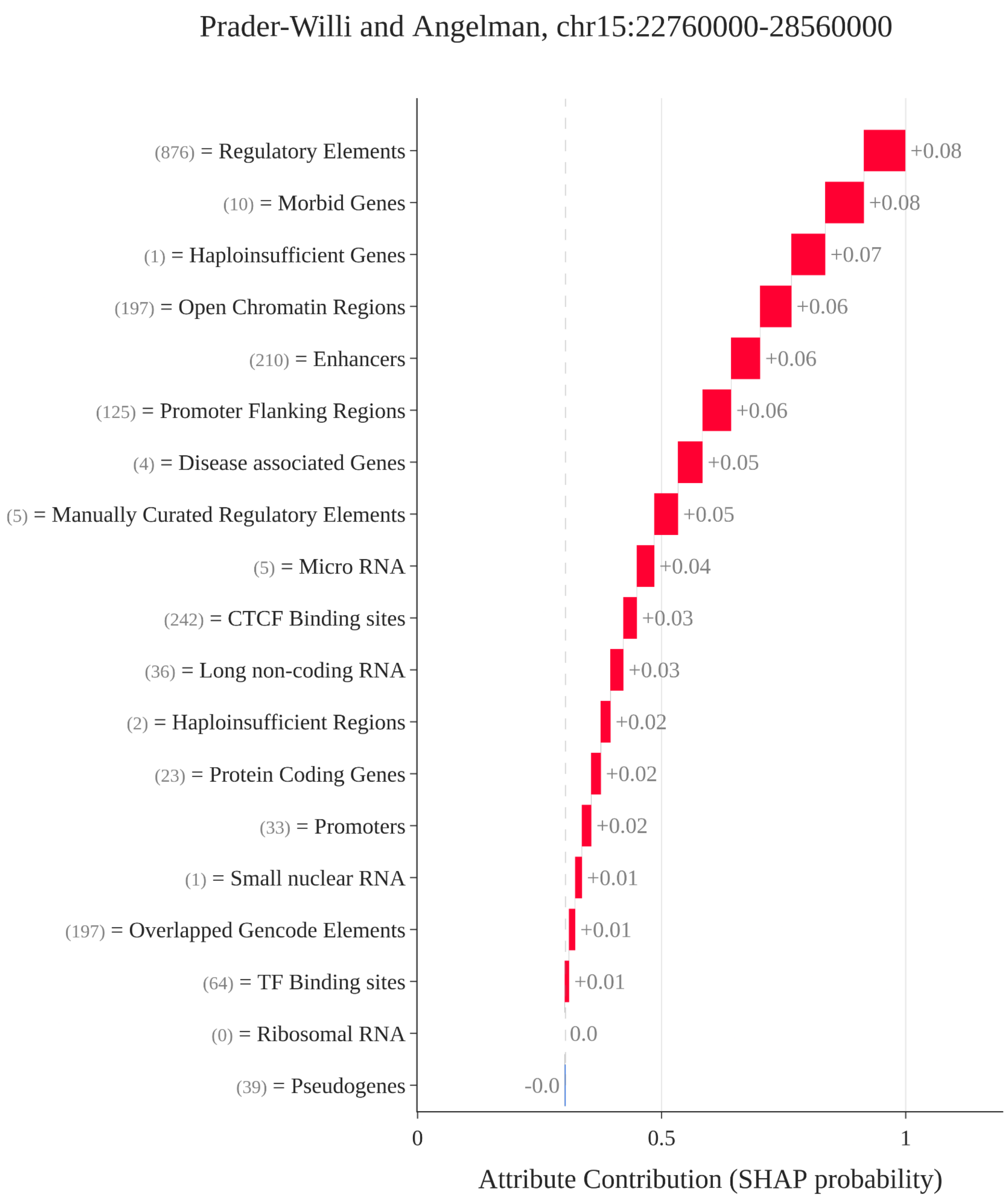

Supplementary Figure S6: Waterfall plot of SHAP value effects of each attribute on the final prediction for a variant responsible for the Prader-Willi and Angelman syndrome. The attributes are sorted by the absolute value of contribution.

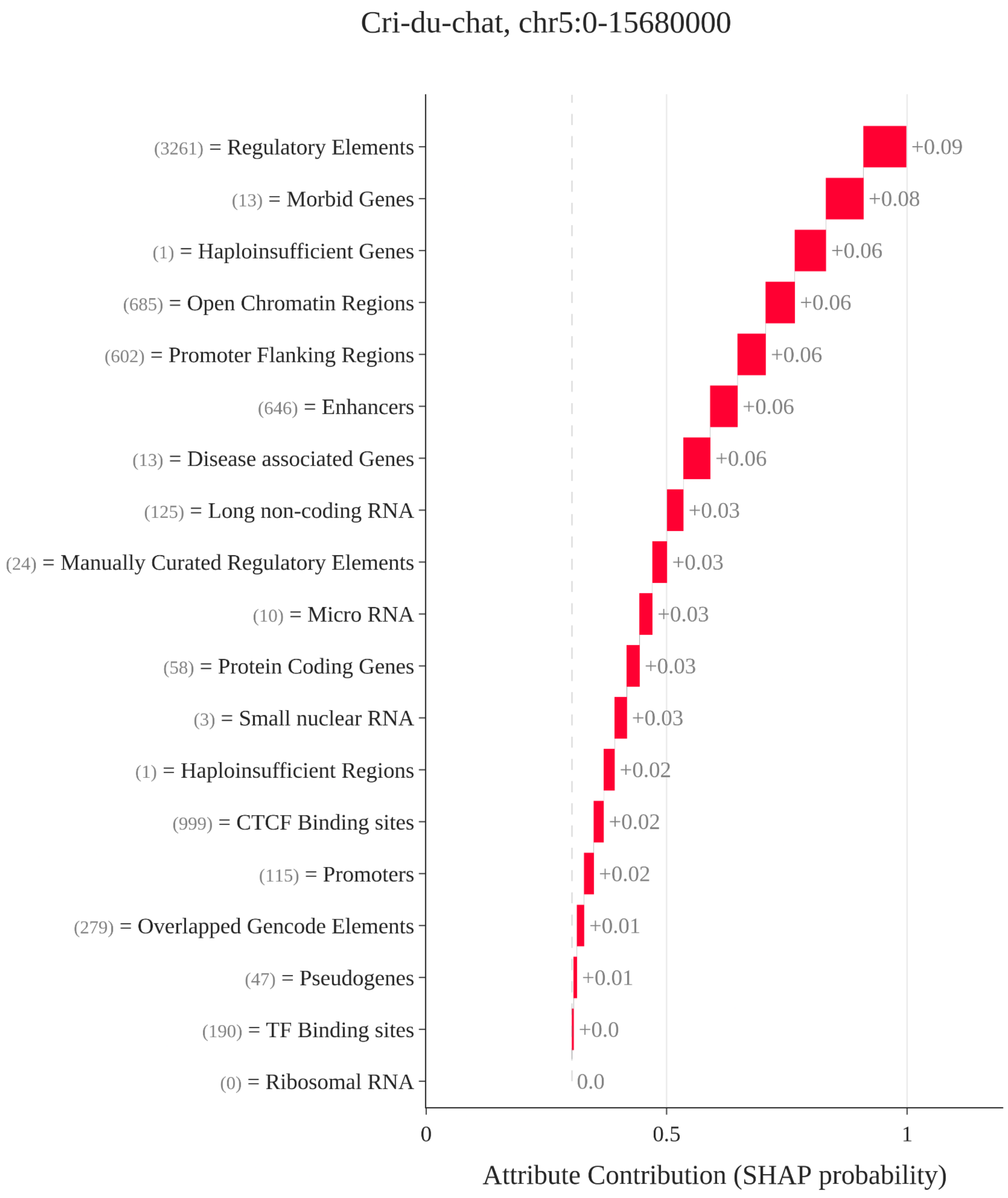

Supplementary Figure S7: Waterfall plot of SHAP value effects of each attribute on the final prediction for a variant responsible for the Cri-du-chat syndrome. The attributes are sorted by the absolute value of the contribution.

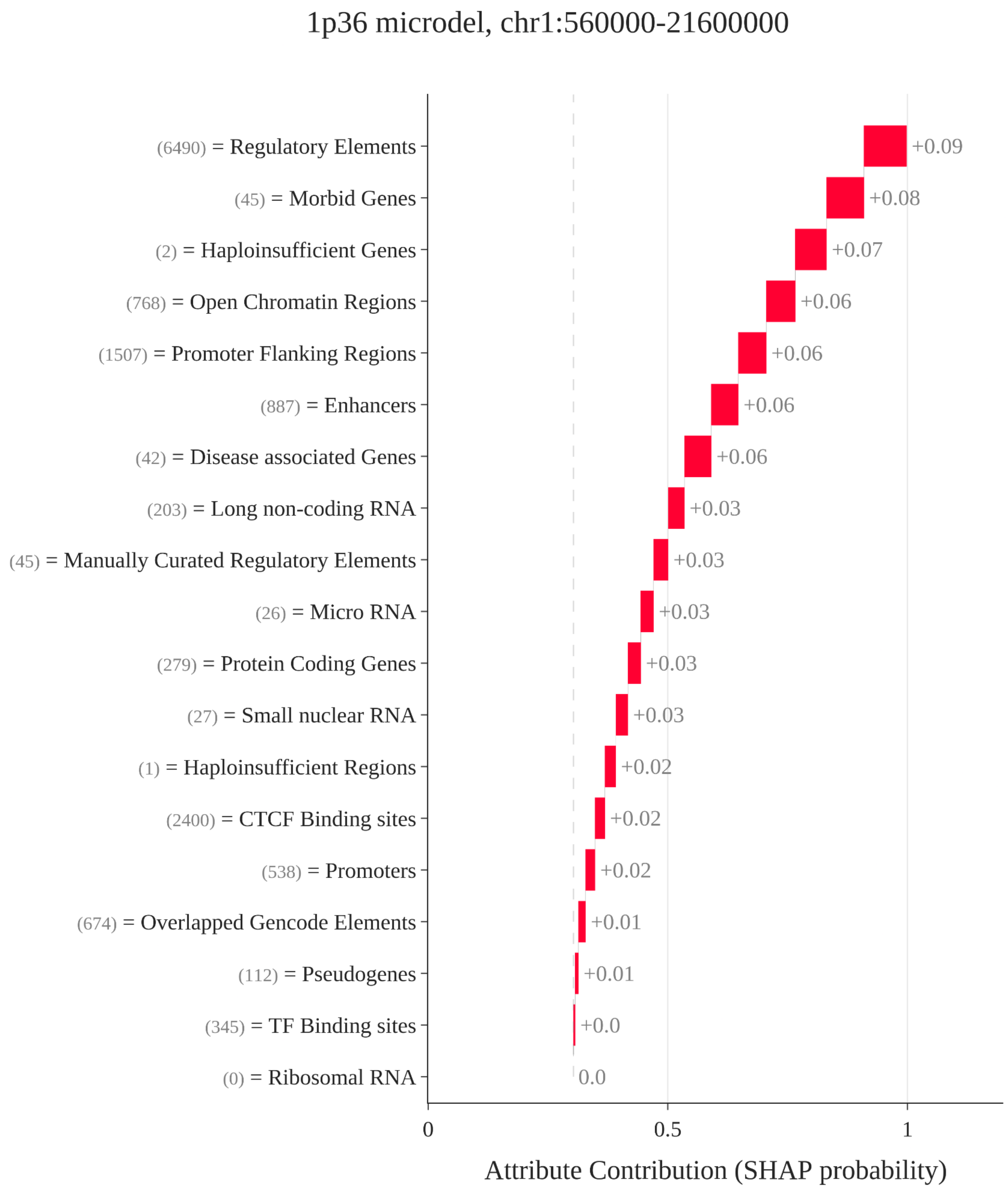

Supplementary Figure S8: Waterfall plot of SHAP value effects of each attribute on the final prediction for a variant responsible for the 1p36 deletion syndrome. The attributes are sorted by the absolute value of the contribution.

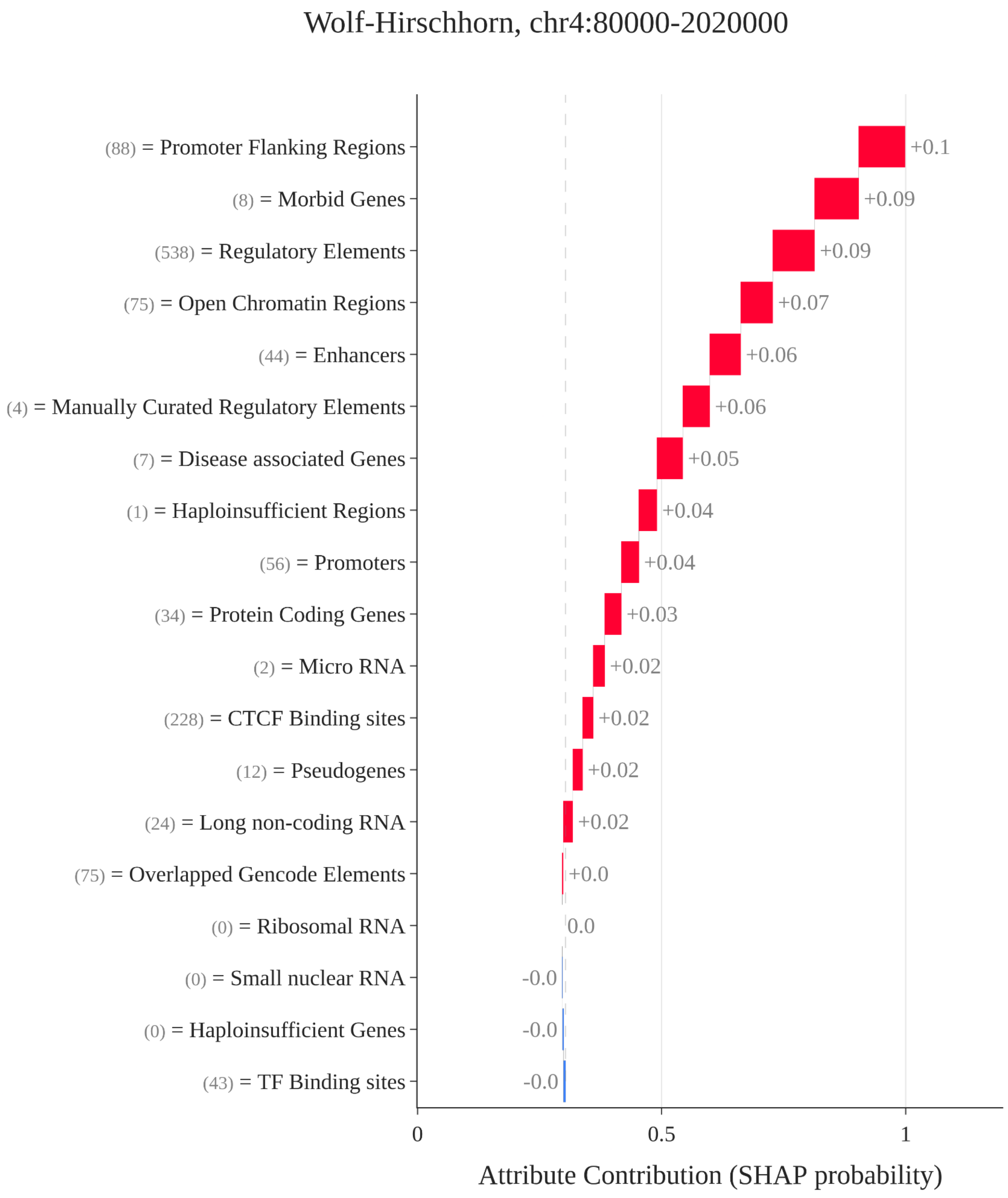

Supplementary Figure S9: Waterfall plot of SHAP value effects of each attribute on the final prediction for a variant responsible for the Wolf-Hirschhorn syndrome. The attributes are sorted by the absolute value of the contribution.

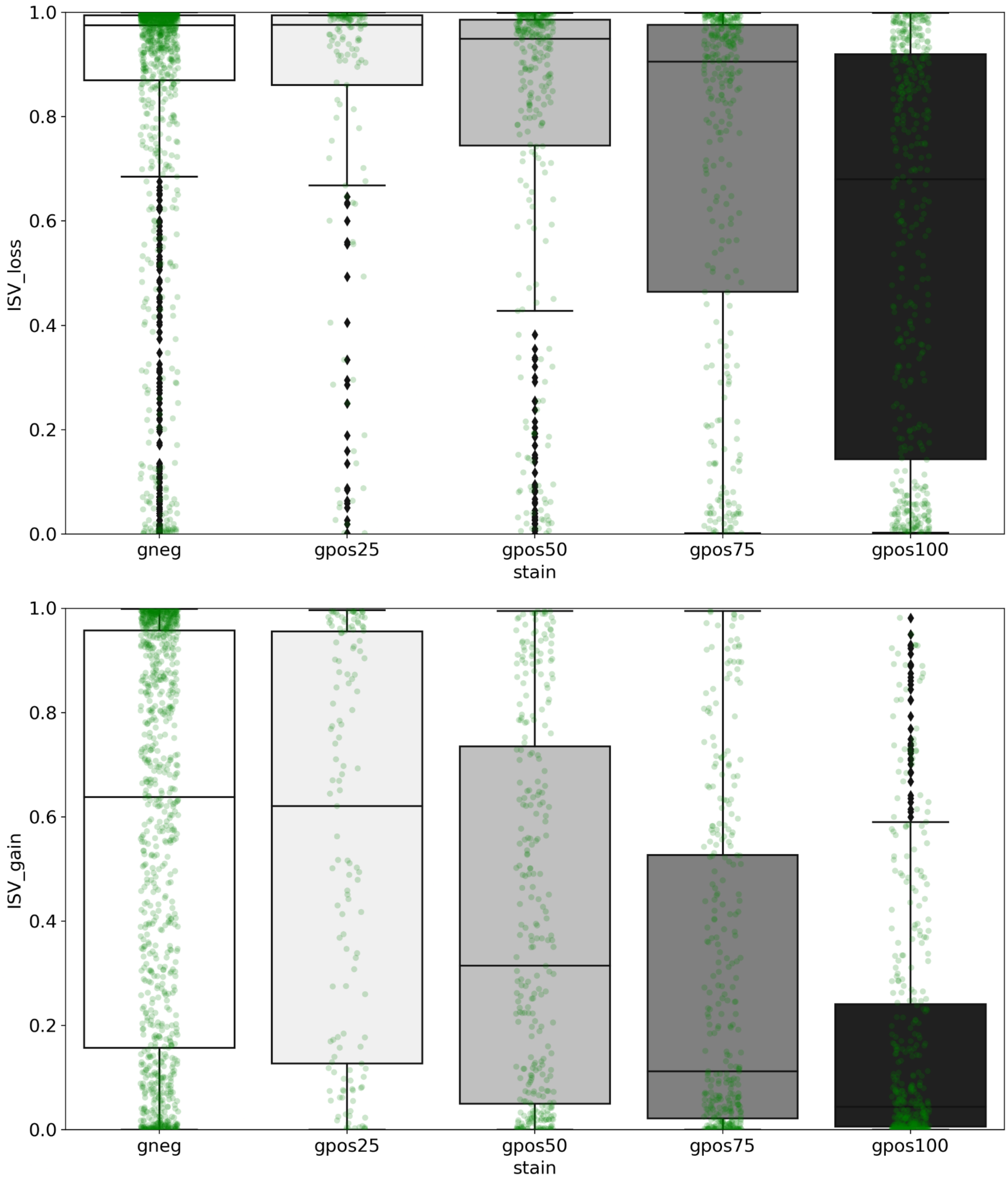

Supplementary Figure S10. : ISV predictions are dependent on the density of GC pairs in the genome. Giemsa stain labels on the x-axis represent 1 Mbp CNVs completely contained within a corresponding stain region. The dataset was downloaded using functions from the circlize package [1], which downloads data from UCSC [2]. The boxplots show that with decreasing GC content (which correlates with biological features of genome organization, such as gene density) the average prediction of pathogenicity decreases. The colors of the boxplots correspond with the stain colors used in the ideogram.

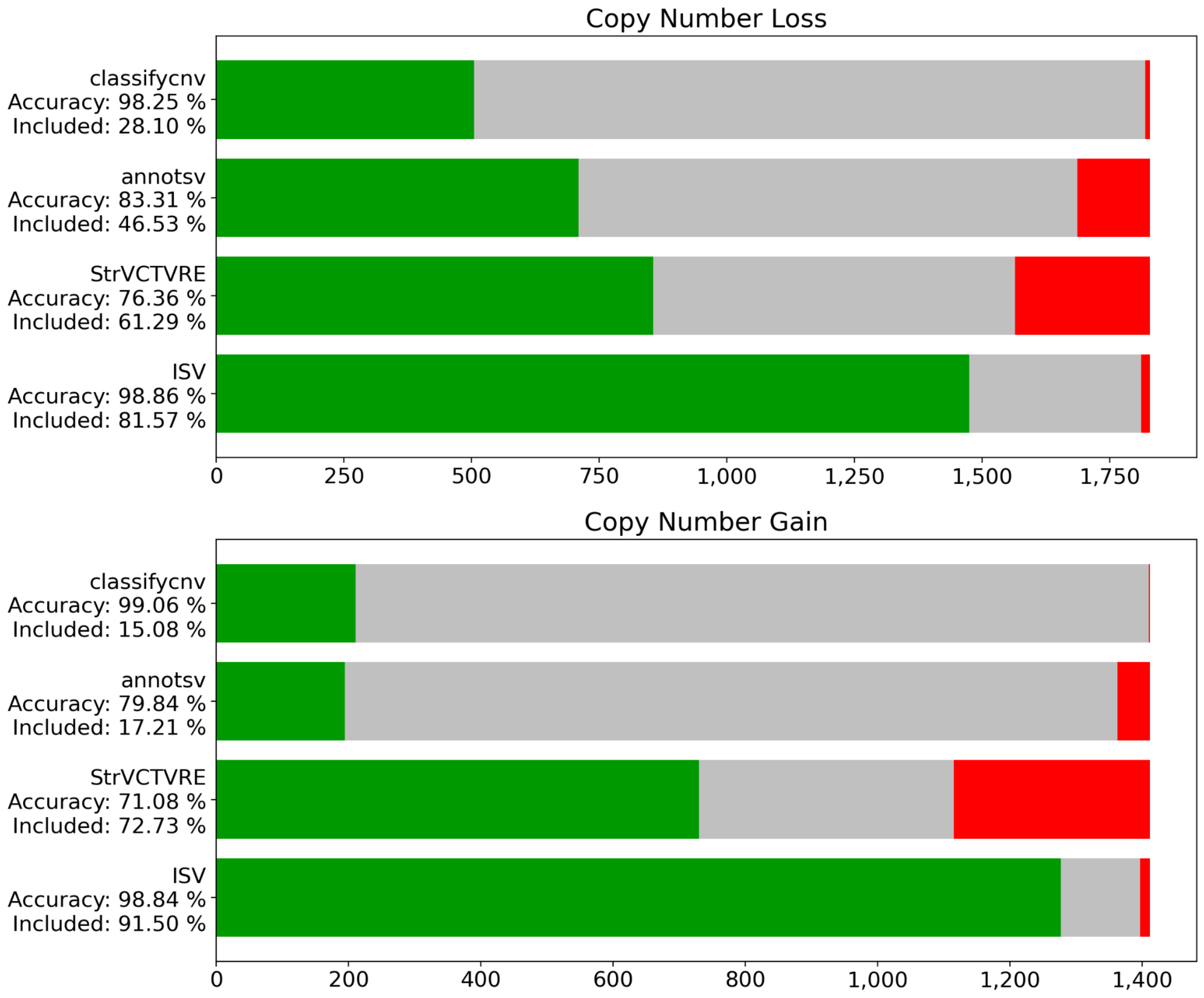

Supplementary Figure S11: Numbers of correct (green), incorrect (red), and uncertain (gray) predictions on the test data. For ClassifyCNV and AnnotSV we treated likely benign as benign and likely pathogenic predictions as pathogenic as opposed to the case represented in Fig. 5 in the manuscript.

Datasets

Datasets derived from ClinVar

Data for training, validation, and basic testing of the method's predictive performance were acquired from the ClinVar database (downloaded 27th April 2021) [3], which represents a shared source of observed genomic variation collected from various studies and individual patients. We extracted and filtered out variants of the "copy number loss/gain" type and with known NCBI36, GRCh37, or GRCh38 inner coordinates. All coordinates were lifted over to the GRCh38 coordinate systems using the UCSC liftover tool [2]. To create the three main datasets we only used variants longer than 1 Kbp and shorter than 5 MBps, labeled as either benign or pathogenic, while duplicates were removed as well as variants with conflicting labels with regard to their pathogenicity. When selecting benign and pathogenic CNVs, the five-tier classification (pathogenic, likely pathogenic, uncertain significance, likely benign, benign) used in ClinVar was used, however, without revision by the authors, therefore there is no certainty that all of them are classified completely in line with the respective ACMG/AMP guidelines [4]. It should also be mentioned here that, although we used this classification as "technical" ground truth, it does not necessarily cover the "biological" truth about the nature and clinical significance of these variants, possibly having a certain effect on the results of our validation study. To create the three main study datasets we divided the remaining CNVs to form the training dataset (70 %), validation dataset (15 %), and testing dataset (15 %).

Beyond these main datasets, and as part of the additional testing datasets, we used some CNVs filtered out from the original ClinVar set to further test the performance of the ISV model. These gave rise to three additional Testing sets. At first, those CNVs which were longer than 5 Mbs (labeled as Testing (>5 Mbs) in Table 1) were filtered out because of their length. The second contained CNVs having multiplicity since ClinVar conveniently offers information regarding the multiplicity of the CNVs. We included heterozygous CNVs deleted or duplicated on one copy of chromosome only for the training and model validation, leaving the rest (homozygous deletions on both chromosomes or CNVs duplicated/amplified twice) for final evaluation (labeled as Testing (multiple) in Table 1). The third additional dataset contained CNVs longer than 1 Kbp, without considering CNV multiplicity, however, containing also CNVs belonging to the classes likely benign, likely pathogenic, and uncertain significance, alongside those labeled as benign and pathogenic from the basic testing set (labeled as Testing (5-tier) in Table 1).

CNVs from gnomAD

The gnomAD database [5], apart from other data, contains CNVs found in sequenced genomes of individuals that were part of various disease-specific and population genetic studies. Individuals known to be affected by a severe pediatric disease, as well as their first-degree relatives were deleted. Some individuals with severe disease may still be included in the dataset, although at a frequency equivalent to or lower than seen in the general population. We evaluated 169100 copy number losses and 49109 copy number gains (gnomAD v2.1). In both cases, the majority of variants occur at population

frequencies lower than 0.05. Due to selection, we expect pathogenic variants to occur at low frequencies, while more frequent variants should be benign or less harmful.

Pathogenic microdeletions and microduplications

To further evaluate the precision of our method to reveal the pathogenicity of CNVs, we manually collected 131 well-established disease-associated microdeletion and 33 microduplication syndromes. Coordinates of these genomic regions were obtained from OMIM and DECIPHER database, and several of them were manually curated to obtain a minimal critical region for each particular disease based on available scientific literature. Descriptions of syndromes, their disease-causing genes, coordinates, and links to syndromes can be seen in Supplementary Table S3.

Association of CNV length with pathogenicity

A model utilizing length as its sole feature is not sufficient, despite the evident tendency of longer CNVs towards pathogenicity even in our validation data set (Supplementary Fig. S12). In both loss and gain CNVs, a simple Logistic regression can be constructed reaching validation accuracies of ~88 %, validation sensitivity, and specificity of 74.9 %, 94.9 % for copy number losses, and 72.8 % and 95.4 % for copy number gains respectively. This is far beyond the performance of the ISV model, which does not explicitly take length into account.

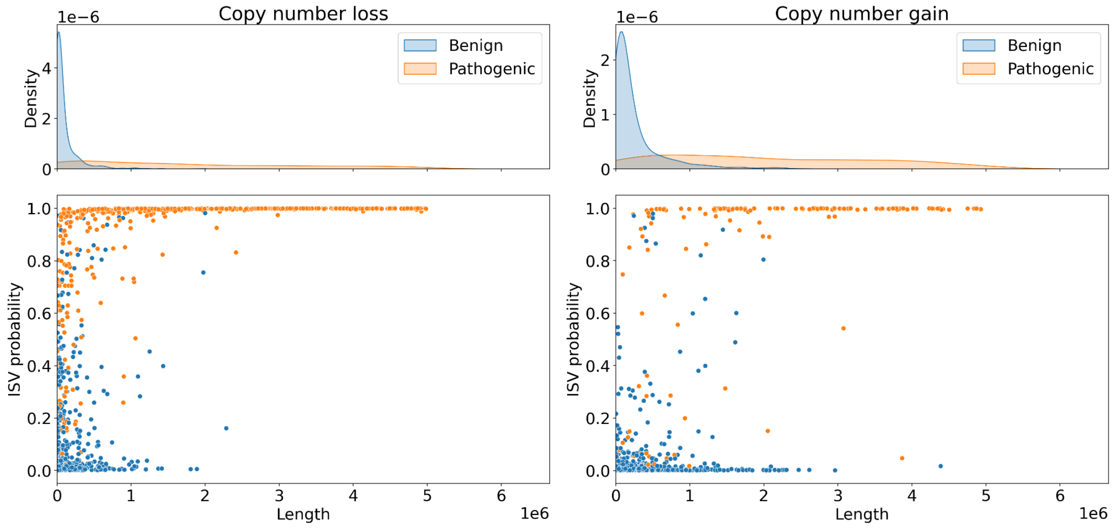

Supplementary Figure 12: Ability of prediction using Length as the only predictor shows that for more reliable predictions other attributes are necessary. The top row shows the distribution of benign and pathogenic variants according to their length. The bottom row shows predictions of ISV against CNV length

Supplementary Discussion

Evaluation of pathogenic microdeletions and microduplications has shown several predictions that did not match expectations. Among deletions, the ISV predicted the loss of Xp22.33 (chrX:791,143-907,140; GRCh38/hg38) as a variant of uncertain significance. However, the region encompasses several distal regulatory elements of SHOX transcription in pseudoautosomal region 1 (PAR1), which deletions were shown to account for approximately 15 % of the patients with Leri-Weill dyschondrosteosis [OMIM #127300] and 2 % of idiopathic short stature [OMIM #300582] [6,7] and they have also been implicated in autism spectrum disorders and related neurodevelopmental conditions [8]. The loss of Yq11.223 (chrY:22,831,278-25,887,782; GRCh38/hg38) was predicted as a variant of uncertain significance, but complete deletion of the AZFc interval of the Y chromosome is the most common known genetic cause of spermatogenic failure [OMIM #415000] [9].

Several duplications had also a different prediction than expected. Since gain of 5q23.2 (chr5:126,776,622-126,837,020; GRCh38/hg38) was shown to cause adult-onset autosomal dominant leukodystrophy (ADLD) [OMIM #169500], at least it should be considered likely pathogenic. However, ISV prediction for copy number gain of this genomic region was likely benign. Coordinates of the region were acquired from DECIPHER database, but the study by Giorgio et al. revealed a 72-kb minimal critical region involving the *LMNB1* gene, required for the development of the disease [10]. When we applied coordinates of the critical region from the study, the ISV prediction

changed to uncertain significance. Moreover, no common breakpoint for ADLD duplication has been observed, and the sizes observed by Giorgio et al. ranged from ~128 Kb to ~475 Kb, which is much larger than the region evaluated by the ISV, suggesting that duplication of the *LMNB1* gene alone is not sufficient to predict CNV as pathogenic. Similar to the previous case, DECIPHER coordinates for the gain of 21q21.2 (chr21:25,880,549-26,171,128; GRCh38/hg38) leading to the early-onset Alzheimer disease [OMIM #104300], was not large enough to predict CNV as pathogenic as it is a region of a single gene APP.

Several X-linked microduplications were predicted as variants of uncertain significance. The coordinates for these duplications (e.g., Xp11.22, Xq22.3, and Xq28) were obtained from the manually curated collection of CNV syndromes in the DECIPHER database. Interestingly, all the mentioned cases of deletions and duplications with non-pathogenic predictions were exclusive for coordinates obtained from DECIPHER, suggesting that some established minimal critical regions in this database are problematic for ISV prediction or based on its genomic content these regions may not be sufficient to cause a corresponding disease phenotype. Although some predictions were missed, the ISV helps to search and merge data from relevant databases in one place that tremendously simplifies the process of variant interpretation and subsequent classification.

During the evaluation phase, we also found room for improvement in the prediction of CNVs encompassing cancer-associated genes. For example, a known proto-oncogene *MYCN* was shown to be amplified in a variety of tumors, most notably neuroblastomas [11]. Based on this evidence, we expected that copy number gain of this region should be pathogenic. However, ISV prediction was likely benign. A similar case was a *VHL*

gene, a known tumor suppressor, that loss is regarded as an initial event for the development of von Hippel-Lindau syndrome [OMIM #193300] associated tumors [12,13], but ISV predicts this CNV as likely benign. These results highlight the limitation of ISV and suggest the need to include additional attributes (CNV overlap with tumor suppressor gene or proto-oncogene) to the ISV prediction process.

References

1. Gu, Z., Gu, L., Eils, R., Schlesner, M. & Brors, B. circlize Implements and enhances circular visualization in R. *Bioinformatics* **30**, 2811–2812 (2014).
2. Kuhn, R. M., Haussler, D. & Kent, W. J. The UCSC genome browser and associated tools. *Brief. Bioinform.* **14**, 144–161 (2013).
3. Landrum, M. J. *et al.* ClinVar: improving access to variant interpretations and supporting evidence. *Nucleic Acids Res.* **46**, D1062–D1067 (2018).
4. Riggs, E. R. *et al.* Technical standards for the interpretation and reporting of constitutional copy-number variants: a joint consensus recommendation of the American College of Medical Genetics and Genomics (ACMG) and the Clinical Genome Resource (ClinGen). *Genet. Med.* 1–13 (2019).
5. Collins, R. L. *et al.* A structural variation reference for medical and population genetics. *Nature* **581**, 444–451 (2020).
6. Benito-Sanz, S. *et al.* A novel class of Pseudoautosomal region 1 deletions downstream of SHOX is associated with Leri-Weill dyschondrosteosis. *Am. J. Hum. Genet.* **77**, 533–544 (2005).
7. Benito-Sanz, S. *et al.* Identification of the first recurrent PAR1 deletion in Léri-Weill dyschondrosteosis and idiopathic short stature reveals the presence of a novel SHOX

enhancer. *J. Med. Genet.* **49**, 442–450 (2012).

8. Tropeano, M. *et al.* Microduplications at the pseudoautosomal SHOX locus in autism spectrum disorders and related neurodevelopmental conditions. *J. Med. Genet.* **53**, 536–547 (2016).

9. Kuroda-Kawaguchi, T. *et al.* The AZFc region of the Y chromosome features massive palindromes and uniform recurrent deletions in infertile men. *Nat. Genet.* **29**, 279–286 (2001).

10. Giorgio, E. *et al.* Analysis of LMNB1 duplications in autosomal dominant leukodystrophy provides insights into duplication mechanisms and allele-specific expression. *Hum. Mutat.* **34**, 1160–1171 (2013).

11. MYCN MYCN proto-oncogene, bHLH transcription factor [Homo sapiens (human)] - Gene - NCBI. https://www.ncbi.nlm.nih.gov/gene?Db=gene&Cmd=DetailsSearch&Term=4613.

12. Montani, M. *et al.* VHL-gene deletion in single renal tubular epithelial cells and renal tubular cysts: further evidence for a cyst-dependent progression pathway of clear cell renal carcinoma in von Hippel-Lindau disease. *Am. J. Surg. Pathol.* **34**, 806–815 (2010).

13. Chew, E. Y. Ocular manifestations of von Hippel-Lindau disease: clinical and genetic investigations. *Trans. Am. Ophthalmol. Soc.* **103**, 495–511 (2005).
